# Metabolite profiling of susceptible and resistant wheat (*Triticum aestivum*) cultivars responding to *Puccinia striiformis* f. sp. *tritici* infection

**DOI:** 10.1186/s12870-023-04313-9

**Published:** 2023-06-01

**Authors:** Manamele Dannies Mashabela, Fidele Tugizimana, Paul Anton Steenkamp, Lizelle Ann Piater, Ian Augustus Dubery, Msizi Innocent Mhlongo

**Affiliations:** 1 Research Centre for Plant Metabolomics, Department of Biochemistry, University of Johannesburg, Auckland Park, P.O. Box 524, Johannesburg, 2006 South Africa; 2 International Research and Development Division, Omnia Group, Ltd, Johannesburg, 2006 South Africa

**Keywords:** LC-MS, Metabolomics, Metabolic reprogramming, *Puccinia striiformis* f. sp. *tritici*, Resistance

## Abstract

**Background:**

*Puccinia striiformis* f. sp. *tritici* (*Pst*) is an economically devasting disease that is prominent in cereal crops such as wheat (*Triticum aestivum*). The fungal pathogen can cause approximately 30–70% losses in crop productivity and yields. *Pst* has become difficult to manage due to its ease of transmission through wind dispersal over long distances, and intercontinental dispersal has been previously reported. The ease of transmission has resulted in further destruction because of new and more virulent strains infecting crops previously resistant to a different strain.

**Results:**

In this study, a liquid chromatography-mass spectrometry-based untargeted metabolomics approach, in combination with multivariate data analytical tools, was used to elucidate the mechanistic nature of the defence systems of a *Pst*-resistant and a susceptible wheat cultivar infected with *P. striiformis*. We also investigated the time-dependant metabolic reconfiguration of infected plants over a four-week period. The untargeted metabolomic analysis revealed a time-course metabolic reprogramming involving phenylpropanoids (majority flavonoids), amino acids, lipids, benzoic acids, TCA cycle intermediates and benzoxazinoids responding to *Pst* infection. Interestingly, the results do not show a linear course for the decrease and increase (up-/down-regulation) of said classes of metabolites, but rather the up- or down-regulation of specific metabolites in response to the pathogen infection. The resistant Koonap cultivar had an abundance of phenolic compounds such as rutin, isoorintin-7-O-glucoside and luteolin-6-C-hexoside-O-hexoside. These compounds showed a decrease over time in control Koonap plants compared to an increase in *Pst*-infected plants. These metabolites were down-regulated in the susceptible Gariep cultivar, which could serve as biomarkers for plant responses to biotic stress and resistance against *Pst*.

**Conclusions:**

Overall, an LC-MS-based metabolomics approach allowed for the metabolic profiling and analysis of the impact of plant-pathogen interactions on the overall plant metabolome and provided a real-time snapshot of the differential significant metabolic perturbations occurring in wheat plants responding to the *Pst* pathogen. The *Pst*-resistant Koonap cultivar showed a rapid accumulation of defence metabolites in response to pathogen infection compared to the susceptible Gariep cultivar. These findings provide insight into the mechanistic biochemical nature of plant-microbe interactions and the prospects of metabolic engineering for improved plant tolerance and resistance to biotic stresses.

**Supplementary Information:**

The online version contains supplementary material available at 10.1186/s12870-023-04313-9.

## Background

Plants are sessile organisms and, as such, are constantly exposed to and must fight off a wide range of evolving pathogenic microorganisms, many of which have been challenging to control due to their relative ease of mobility, (re-)emergence or rapid genetic evolution [1]. Over the years, plants have evolved strategic defence mechanisms such as the multi-layered innate immune response relating to pathogen/microbe-associated molecular pattern (P/MAMP)-triggered immunity (P/MTI) and effector-triggered immunity (ETI) to reduce or prevent the proliferation of pathogenic attacks [2–4]. The basis of PTI/ETI-induced plant immune responses is modelled on the fundamental yet overarching principle of signal perception and subsequent transduction [2], thus leading to the activation and expression of defence-related genes and biosynthesis of antimicrobial compounds. In addition, ETI offers the advantage of a rapid response through a localised hypersensitive response (HR) in which infected tissue undergoes programmed cell death, thus reducing the spread of the infection and pathogen proliferation to fresh/uninfected tissue [5, 6].

HR is correlated to several changes in the affected plant cells, which include the strengthening of structural barriers, production of phytoalexins, the accumulation of pathogenesis-related (PR) proteins, and the biosynthesis of lytic enzymes [7]. These changes generally lead to the induction of systemic acquired resistance (SAR) through salicylic acid (SA) or induced systemic resistance (ISR) through jasmonic acid-ethylene (JA/ET)-mediated signalling pathways and *PR* gene expression to generate a broad spectrum of plant resistance [8]. The phytohormones, namely SA, JA and ET, interact synergistically and antagonistically to regulate plant defence responses [9, 10] resulting in prolonged resistance. Resistance is especially significant in high priority cereal crops such as wheat against diseases including stripe rust.

Wheat is one of the most important cereal crops globally and the second most cultivated crop after maize [11]. Most of the wheat production is channelled towards human consumption; some uses include beverage, starch and straw production, with occasional uses in animal feed and bio-fuel production [12]. Wheat production has seen a steady decline by hectarage in the last three decades and has continued to fall since [11, 13]. Among many factors, poor soil fertility and high pH, climate change, poor cultivar choices and environmental stressors such as disease breakouts, including yellow wheat stripe rust, have contributed to the observed losses in production rates, quality and yields [14]. Yellow wheat stripe rust is an economically important disease caused by the fungus *Puccinia striiformis* f. sp. *tritici* (*Pst*) [15]. *Pst* is an obligate biotroph and, therefore, can infect the host plant at different growth stages and feeds on live wheat plants for nutrition acquisition [16].

Yield losses of 10–100% have been reported due to the pathogen’s interference with the plant’s primary metabolism through depletion of the host’s energy reserves for its growth. Subsequently further colonisation and reproduction interfere with the plant’s photosynthetic capacity leading to induced chlorosis and necrosis. [16, 17]. Carmona et al. [16] explained that the magnitude of the yield losses from *Pst* is dependent chiefly on the degree of resistance of the various cultivars against *Pst* along with the growth stage of the plant at the disease onset, with high yield losses occurring in susceptible cultivars. The emergence of new and more aggressive strains of *Pst* has increased the wheat industry’s concerns at large. Efforts have been made to control the progression of *Pst* in recent years, for example the application of foliar fungicides has long been an essential component of *Pst* control mechanisms and is still in use to date [16]. However, agrochemicals have been met with criticism for the negative impact on the environment [18–20]. Alternatively, breeding for *Pst*-resistant cultivars offers a pre-emptive, efficient and sustainable method of control for the future and should be the focus of breeders [17]. The release of host-plant resistant wheat cultivars has been, for the most part, a reliable, environmentally-friendly and cost-effective strategy for *Pst* control [17].

Many studies have focused on the genomic variability of resistant wheat cultivars compared to their susceptible counterparts to develop and introduce new resistance genes for breeding strategies [1, 15]. For instance, a transcriptomics approach to understanding the underlying mechanisms characteristic of wheat resistance to stripe rust revealed transcriptomic reprogramming in cultivars through the up- and down-regulation of approximately 6 000 unigenes, showing candidate genes as likely contributors to plant resistance against *Pst* infection [21, 22]. More recently, Kim et al. [23] performed a metabolomics profiling of *Pst-*resistant wheat cultivars in combination with transcriptomic analysis to investigate the biochemical mechanisms associated with leaf rust resistance. The study revealed 45 metabolites flagged as promising biomarkers for resistance and pointed to the phenylalanine metabolic pathway as a contributor to conferring leaf rust resistance by increasing the levels of secondary defence metabolites including flavonoids. On the other hand, increased levels of primary metabolites such as amino acids were also observed and was suggested to confer resistance to leaf rust through alanine, aspartate and glutamate metabolism. Studies suggest that metabolites and metabolic pathways can serve as gateways to screen targeted compounds for disease resistance and crop improvement. The current study investigates the time-dependent changes in the metabolic profiles of both a resistant and susceptible wheat cultivar responding to *Pst*. The study also aims to elucidate the metabolic reprogramming in wheat during the defence response and identify potential *Pst* resistance biomarkers.

## Results

### *Pst* infection and symptom development

The development of symptoms in *Pst*-inoculated plants was monitored from 14 days post-inoculation for three consecutive weeks. Symptom monitoring and evaluation was carried out with reference to [24]. The infected variants of both the susceptible Gariep and resistant Koonap cultivars showed *Pst* infection (Fig. S1). Upon closer inspection, Koonap plants developed notable portions of severe leaf chlorosis surrounding the areas of infection, with signs of necrosis and further surrounded by healthy green leaf areas. On the other hand, the Gariep cultivar showed mild chlorosis with no signs of necrosis at the sites of infection; additionally, the susceptible cultivar showed further yellowing of entire leaf areas due to *Pst* infection compared to control plants (Fig. S1). Furthermore, visual examination of sporulation revealed the development of large and more pronounced spores on the Gariep cultivar compared to the Koonap counterpart. Moreover, spore development was restricted to portions of the leaf surface area in the *Pst*-infected Koonap cultivar compared to the general spread of spores along the length of the leaf area, showing a greater rate of infection and pathogenesis in the *Pst*-infected Gariep Cultivar.

### Metabolic profiling of *pst*-induced perturbations in resistant and susceptible wheat cultivars

Wheat cultivars were selected to elucidate the responsive mechanisms of the susceptible variety (Gariep) to *Pst* compared to its resistant counterpart (Koonap) at a metabolic level. Previous studies have reported metabolic perturbations in plants due to plant-pathogen interactions. In the current study, methanolic extracts of *Pst*-treated and untreated Koonap and Gariep wheat cultivars were analysed on an LC-MS system to acquire chromatographic and MS profiles of the samples. Chromatographic separation was useful for separating the chemically and structurally diverse classes of molecules making up the plant metabolomes of extracted samples and generated base peak intensity (BPI) MS chromatograms. Visual observation of the BPI MS chromatograms shows some quantitative (accumulation/peak intensities) and qualitative (presence/absence) variations in the metabolic profiles of *Pst*-treated and untreated samples (Fig. S1 to S4). The clear differences observed point to the alterations in the cellular metabolism and overall metabolomes of the plants under investigation. However, BPI MS chromatograms present only an overview of the apparent metabolic reprogramming and, as such, the detailed reconfigurations observed were further investigated by multivariate data analysis (MVDA) tools as further described below.

### Multivariate data analysis

**Fig. 1:**
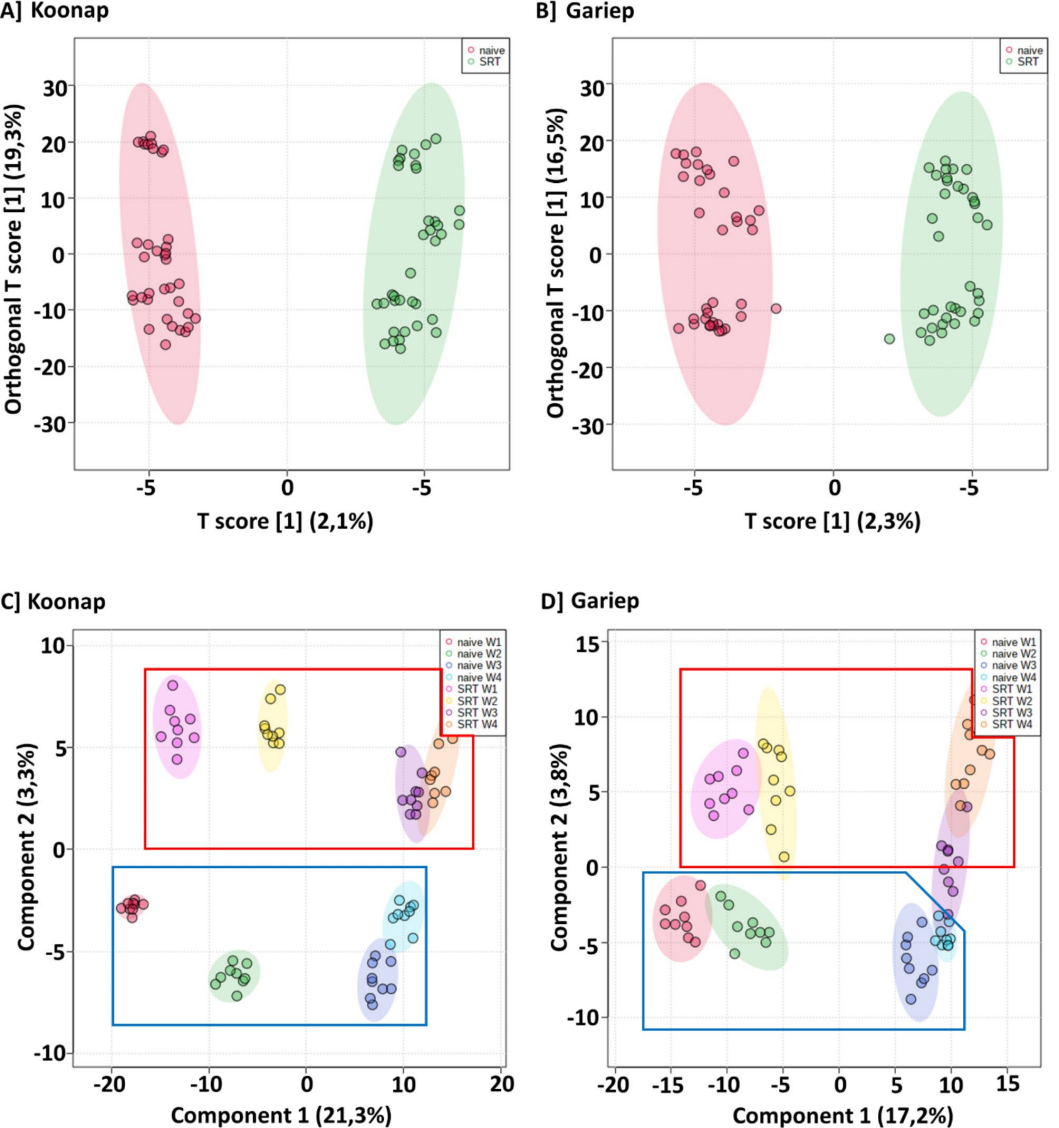
Computed orthogonal partial least squares discriminant analysis (OPLS-DA) and partial least squares discriminant analysis (PLS-DA) models. (**A**) and (**B**) are computed OPLS-DA models showing the separated metabolic features of the control (red) and *Pst-*infected (green) samples of Koonap and Gariep wheat cultivars, respectively. (**C**) and (**D**) further show time-dependent metabolic changes in both control (control in blue demarcation) and infected (SRT in red demarcation) samples, starting from week 1 (W1) post-inoculation to week 4 (W4) post-inoculation, left to right. The OPLS-DA and PLS-DA models show differential separation of data based on pathogen infection as well as plant development over time as an indication of pathogen-induced metabolic reprogramming. The data projected above were median-normalised, log transformed and *Pareto-*scaled in MetaboAnalyst for correlation and predictability scores of *R*2 = 0.921 and *Q*2 = 0.600 (**A**); *R*2 = 0.609 and *Q*2 = 0.256 (**B**); *R*2 = 0.944 and *Q*2 = 0.702 (**C**); *R*2 = 0.890 and *Q*2 = 0.528 (**D**)

Due to the enormous amounts of data generated from metabolomics analysis and combined with the complexity of the data, a visual inspection of BPI MS chromatograms does not provide adequate representation of all the apparent differences in the metabolic features of the samples. As such, a chemometrics-based statistical analysis was used to deconvolute the data and extract trends complementary to the observed variations in Fig. S2 to S4 to paint a clearer picture of significant metabolic features associated with metabolic reprogramming due to *Pst* infections. For this purpose, an orthogonal partial least squares-discriminant analysis (OPLS-DA) was performed to reveal the data structures and underlying patterns (Fig. 1A and B). OPLS-DA is a predictive supervised binary classifier that collects and categorises data from a two-sample group comparative method i.e., sample classification based on *a priori* class information (treated vs. control). Evaluation of the computed OPLS-DA models reveal clear treatment-based separation of control-untreated (control) samples from *Pst*-infected/stripe rust-treated (SRT) samples from the Koonap and Gariep cultivars as illustrated in Fig. 1A and B respectively. These observed separations thus indicate metabolic differences in the *Pst*-infected and control samples, suggesting a metabolic reprogramming due to pathogen infection.

Further evaluation of the changes in the plant metabolome post-*Pst* inoculation was performed over a period of four weeks, and the data from the LC-MS analysis was used to compute PLS-DA models to visualise the time-dependent metabolic reconfiguration of infected plants compared to their uninfected (control) counterparts. The resulting PLS-DA models (Fig. 1C and D) revealed time-dependant variations in the metabolic profiles of the plants as also seen in the BPI chromatograms in Fig. S3 and Fig. S4, thus showing the evidence of an altered metabolism through quantitative and qualitative variations in the peak populations of Gariep and Koonap control vs. infected samples, respectively, over time. The observation gives insights into the underlying biochemistry of the plants’ response to infection as well as the metabolic reconfigurations occurring during plant development. OPLS-DA further provides the advantage of selecting significant discriminatory biomarkers from separated datasets. In this regard, signatory biomarkers are essential for pinpointing specific metabolites responsible for the observed differences in the separations from the OPLS-DA models.

Variable importance projection (VIP) scores were computed to identify, characterise and statistically confirm and validate the selected biomarkers (Fig. S5), and only variables with a score of > 1 were considered as significant. The VIP scores were generated from a dataset of metabolites with reference to Table S1 and putatively annotated using chromatographic and spectral information in combination with the untargeted metabolomics software MS-DIAL as described in [25]. The selected discriminatory metabolites were differentially up-/down-regulated in the control plants compared to *Pst*-infected plants. For instance, infection of the Koonap cultivar resulted in the up-regulation of phenolic metabolites such as rutin, luteolin-6-C-hexoside-O-hexoside, kaempferol-3-O-glucoside, luteolin-C-hexoside-C-pentoside and apigenin C-hexoside-C-pentoside (Fig. S5A). On the other hand, *Pst*-infected Gariep cultivars saw a down-regulation of phenolic compounds including hordatine-C-hexoside, rutin, luteolin-C-hexoside-C-pentoside, vitexin-2’’-O-rhamnoside, kaempferol-3-O-glucoside and 3-feruloyl quinic acid (Fig. S5B). Following the global metabolite annotation, a relative quantification and interactive heatmap analysis was performed to further explore the observed infection-related metabolic reconfigurations in the wheat cultivars. Heatmaps for Koonap samples (data not presented) showed relative differential accumulation of metabolites in the control and *Pst*-infected plants (control vs. SRT) from different classes of metabolites including flavonoids, fatty acids, amino acids, organic acids, indole and indole derivatives. This observed differential regulation of metabolites serves as an indication of the metabolic reprogramming induced by *Pst* infection on the metabolome of the plants.

### Time-dependent metabolic reprogramming in *pst*-infected wheat cultivars

**Fig. 2:**
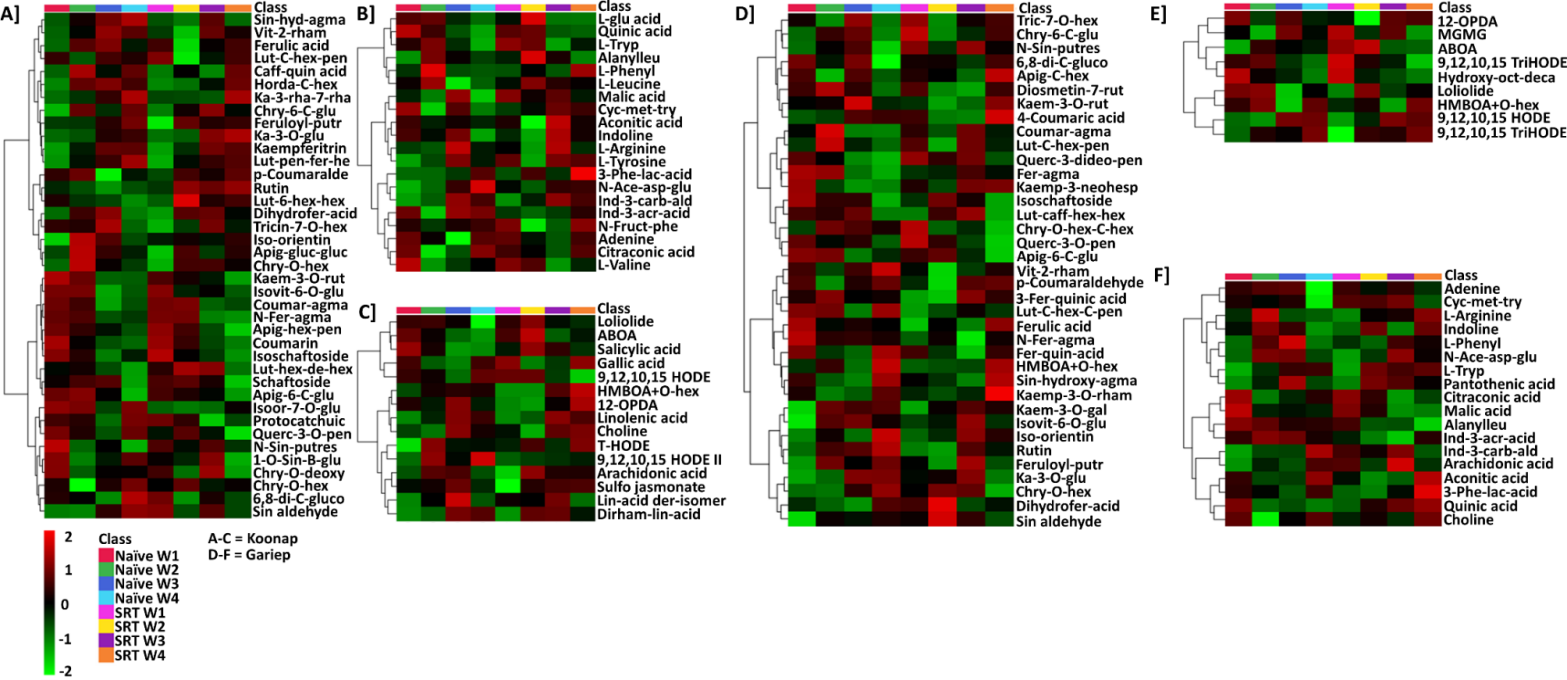
**A-C**: Interactive heatmap analysis of profiles and quantitative distribution of tentatively annotated metabolites in the control vs. *Pst*-infected Koonap cultivar. Figures **A** (flavonoids), **B** (amino acids, organic acids and indoles and derivatives) and **C** (lipids/fatty acids and benzenoids) show the relative quantification of annotated metabolites detected in Koonap samples over a four-week timepoint following infection. **D-F**: Interactive heatmap analysis of profiles and quantitative distribution of putatively annotated metabolites in the control vs. *Pst*-infected Gariep cultivar. Figures **D** (flavonoids), **E** (lipids/fatty acids and benzenoids) and **F** (amino acids, organic acids, indoles and derivatives) describe the relative quantification of annotated metabolites detected in Gariep samples over a four-week timepoint following infection. Up-regulated metabolites are shown in red whereas down-regulated metabolites are indicated by a green rating on the heatmap, while black shading shows no changes in the metabolite concentrations. The data projected above were median-normalised, log transformed and *Pareto-*scaled in MetaboAnalyst. The heatmap analysis thus showed the time-dependent changes in metabolic profiles of wheat plants due to pathogen infection compared to untreated plant samples

A quantitative evaluation of the distribution of annotated metabolites across the different timepoints between control and *Pst*-infected plants was done based on an interactive heatmap analysis in order to project the impact of pathogen infection on the primary and secondary metabolism, and the apparent plant response over time. Additionally, the response of each annotated metabolite with a VIP score of > 1 was determined and included a range of metabolites such as flavonoids, amino acids, fatty acids, indoles and organic acids. The interactive heatmap for Koonap samples (Fig. 2A-C) and Gariep samples (Fig. 2D-F) showed differential regulation of metabolites over the four-week period from the pathogen-infected samples compared to the control samples.

**Fig. 3:**
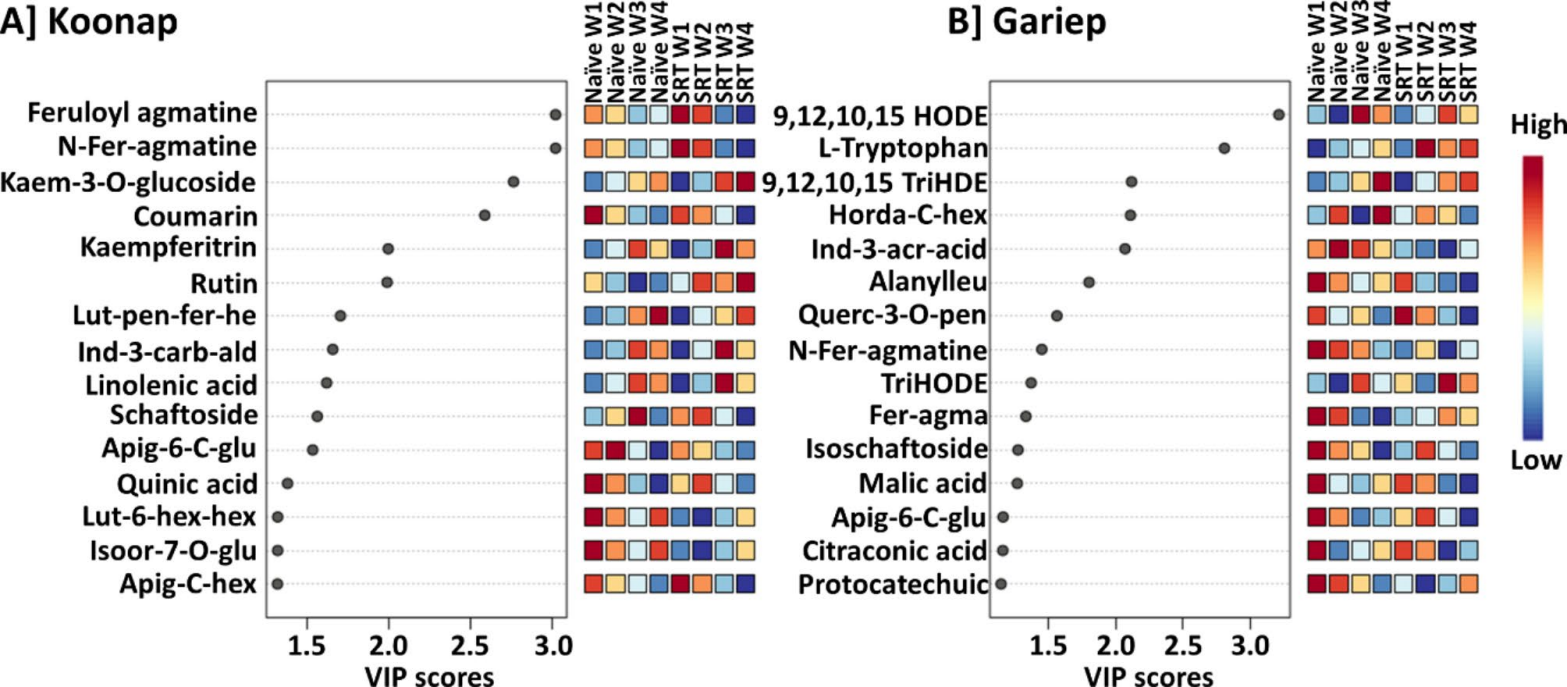
VIP score-plots derived from the PLS-DA analysis. The VIP scores display discriminant features in *Pst*-infected plant samples (SRT) compared to untreated (control) samples for Koonap (**A**) and Gariep (**B**) over a period of four weeks post-inoculation. Selected metabolites (VIP score ≥ 1) in infected plants were compared to those in control plants at the given time point. The figures show differential accumulation of significant metabolites in infected plants compared to control counterparts in correlation to the plants’ developmental stages. Some metabolites increased over time while other decreased. Furthermore, *Pst* infection also induced the up- and down-regulation of metabolites as compared to untreated counterparts. The data projected above were median-normalised, log transformed and *Pareto*-scaled in MetaboAnalyst

The VIP scores (Fig. 3) further show the quantitative distribution and perturbations of the most significantly changed metabolites due to *Pst* infection over time. Selected metabolites with VIP scores of > 1 also present potential biomarkers for the plant response to pathogen infection. These metabolites included, but not limited to, *N-*feruloyl agmatine, coumarin, apigenin-6-C-glucoside and isoorintin-7-O-glucoside, which decreased over the four-week time period in both infected and control Koonap plants, while other metabolites such as kaempferitrin, indole-3-carboxaldehyde, linoleic acid and feruloyl putrescine showed an increased accumulation. Interestingly, rutin, isoorintin-7-O-glucoside and luteolin-6-C-hexoside-O-hexoside showed a decrease over time in control Koonap plants (Fig. 3A) compared to a gradual increase in *Pst*-infected plants which could point to their roles in plant response to biotic stress. Moreover, most significant metabolites from the Koonap samples were flavonoids, which coincides with findings reported by [25]. On the other hand, fewer flavonoids (and other phenolic compounds) made up the significantly altered metabolites in Gariep samples (Fig. 3B), which consisted of a diverse profile of compounds including organic acids, amino acids, indole derivatives and fatty acids. The Gariep cultivar naturally composed of fewer specialised secondary metabolites (flavonoids and other phenolic compounds) compared to the Koonap cultivar [25], a phenomenon displayed from the findings in Fig. 3B, wherein mostly primary metabolites were the most significantly changed metabolites, indicating the reprogramming of the primary metabolisms as compared to the reprogrammed secondary metabolism in the Koonap cultivar. The lack thereof, of an enhanced secondary metabolism in Gariep renders the plant susceptible to *Pst*, with a slower defence response to the infection compared to the resistant Koonap cultivar as indicated by the range of significantly changed metabolites. Therefore, an induced secondary metabolism can improve the resistance of the cultivar. An induced resistance in plants (also known as an induced systemic resistance-ISR), can be adopted through a mechanism called plant priming, in which plants are pre-inoculated with microbes (plant growth-promoting rhizobacteria-PGPR) or natural compound (biostimulants) essential from improving plant defence and tolerance against biotic and abiotic stress [4, 20].

To further evaluate the impact of *Pst* infection on the metabolome of the plants, metabolic pathway analysis (MetPA) was performed using matched IDs of putatively annotated metabolites according to [47]. MetPA is a software programme that integrates univariate and multivariate data analysis methods and pathway topology analysis to generate an overview (identification and visualisation) of the significant and most impactful pathways linked to the specified metabolomic data. The pathways show Kyoto Encyclopedia of Genes and Genomes (KEGG)-matched metabolites in red while unmatched metabolites are indicated in blue. Matched metabolites are integrated to show the interconnection of the metabolites within the metabolic pathways using advanced metabolic pathway data from the KEGG database. KEGG is an integrated computer model used to represent broad biological data in the form of molecular interaction and reaction pathways [26]. Pathway analysis is thus essential for enhanced visualisation of metabolomics data by considering the impact of subtle changes in the concentrations of certain metabolites. Here, the significant pathways are determined by a lower *p*-value on the log10(p) scale, while the impactful pathways are displayed according to their pathway impact factors (Fig. S6A and B).

**Fig. 4:**
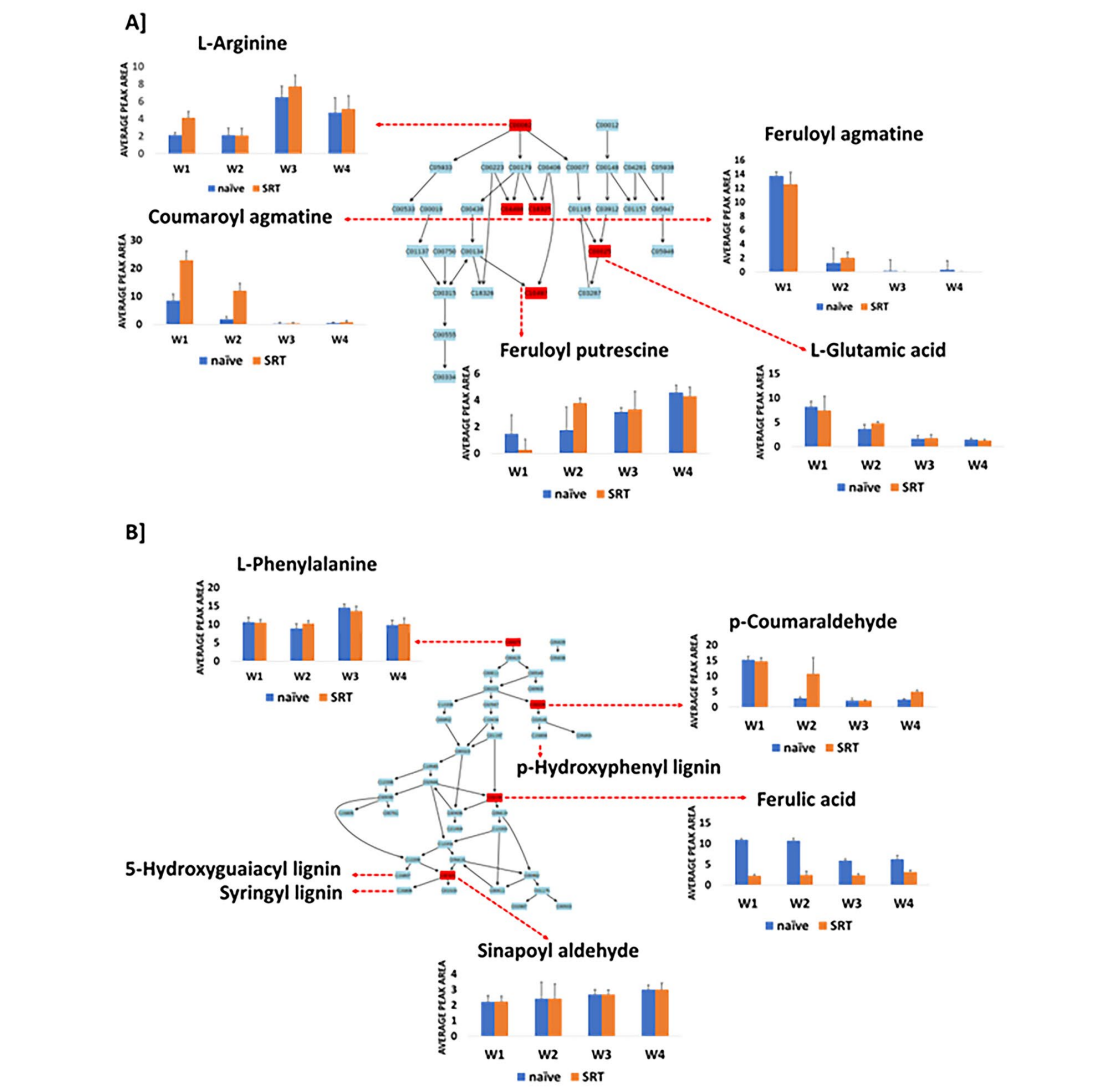
A summary of metabolic pathways analysis generated in MetPA showing significant and impactful pathways in the Koonap cultivar. The diagrammatic illustration shows the topological characteristics of the impacted pathways in **(A)** and **(B)** representing the arginine and proline metabolism pathway as well as the phenylpropanoid pathway respectively, which were the most significantly impacted pathways due to *Pst-*infection in the Koonap cultivar based on the degree of matched annotated metabolites. The bar graphs show the relative quantification of the matched metabolites in the control (blue) and *Pst*-infected (orange) Koonap samples. The matched metabolites from the pathways are as follow: C00062 = L-arginine; C04498 = coumaroyl agmatine; C18325 = feruloyl agmatine; C00025 = L-glutamic acid; C10497 = feruloyl putrescine; C00079 = L-phenylalanine; C05503 = p-Coumaraldehyde; C01494 = ferulic acid; C02325 = sinapoyl aldehyde

**Fig. 5:**
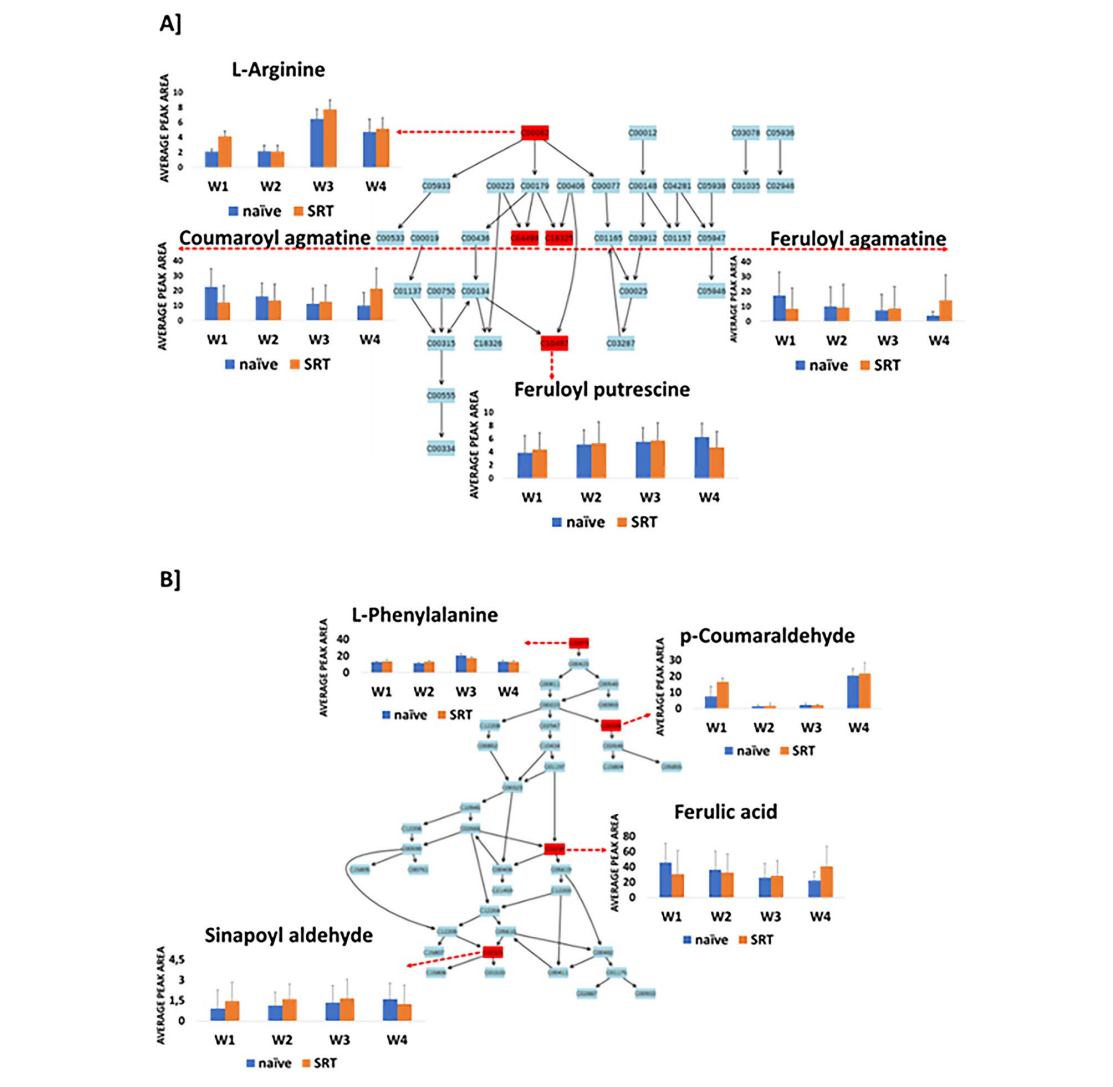
A summary of metabolic pathways analysis generated in MetPA showing significant and impactful pathways in the Gariep cultivar. The diagrammatic illustration shows the topological characteristics of the impacted pathways in **(A)** and **(B)** representing the arginine and proline metabolism pathway as well as the phenylpropanoid pathway respectively, which were the most significantly impacted pathways due to *Pst-*infection in the Gariep cultivar based on the degree of matched annotated metabolites. The bar graphs show the relative quantification of the matched metabolites in the control (blue) and *Pst*-infected (orange) Gariep samples. The matched metabolites from the pathways are as follow: C00062 = L-arginine; C04498 = coumaroyl agmatine; C18325 = feruloyl agmatine; C00025 = L-glutamic acid; C10497 = feruloyl putrescine; C00079 = L-phenylalanine; C05503 = p-Coumaraldehyde; C01494 = ferulic acid; C02325 = sinapoyl aldehyde

The arginine and proline metabolism pathways are essential in primary metabolism, and central in the biosynthesis of the two amino acids from glutamate. This pathway also feeds into the secondary metabolism where arginine is further metabolised to produce the cinnamamides including coumaroyl agmatine, feruloyl putrescine and feruloyl agmatine (Fig. 4A). This observation thus indicates the far-reaching impacts of pathogen (*Pst*) infection on the plant, which causes metabolic reconfigurations in both the primary and secondary metabolism as further shown by the impacted phenylpropanoid pathway (Fig. 4B). Similar results were observed from the Gariep samples, where *Pst* infection induced metabolic reprogramming through both the primary and secondary metabolism, significantly impacting the arginine and proline metabolism, and the phenylpropanoid biosynthesis pathway (Fig. 5). The impacted pathways are essential to produce specialised secondary metabolites commonly associated with plant defence responses. This thus indicates redirected priorities in the plant metabolism geared towards the enhancement of defence against pathogen infection.

Overall, the infection of both Koonap and Gariep plants led to metabolic reconfigurations affecting the primary and specialised secondary metabolism through the up-/down-regulation of specified metabolites. Koonap samples had on average a higher accumulation of phenolic compounds, particularly flavonoid glycosides and other flavonoid conjugates in both *Pst*-infected and untreated samples. Additionally, a significant difference in metabolite accumulation was observed in infected plants compared to untreated plants (Fig.S2) and over time (Fig. S3). On the other hand, major differences were not observed on the chromatograms for the Gariep samples (Fig. S3 and S4), however, further statistical analysis revealed the detailed metabolic reconfigurations in *Pst*-infected samples compared to control controls.

## Discussion

Wheat stripe rust is an economically important disease caused by the obligate biotrophic basidiomycete fungus *Puccinia striiformis* f. sp. *tritici* (*Pst)*, which has become a considerable threat to wheat production around the world, with up to 70% reduction in yields in infected fields [25, 27, 28]. *Pst* depends on nutrient availability from the host plant to complete its life cycle which occurs continually throughout the growing season, and extraction of nutrients occurs at the plasma membrane of the infected host where the fungus injects nutrient transporters [29]. Ultimately, extraction of nutrients is followed by secretion of effector proteins which may cause further nutrient reallocation from the healthy to the infected tissues, subsequently causing a metabolome perturbation that the host plant undergoes for a metabolic reconfiguration to mitigate the deleterious effects of the pathogen infection [29].

The biotrophic nature of *Pst* requires constant nutrient supply from the host for energy production and the maintenance of biosynthetic pathways for spore production [16]. Plants have thus evolved intricate defence systems such as the hypersensitive response (HR) [6, 30]. The HR induces rapid cell death at the site of infection as a means of a defence response against pathogens, and this intrinsic response depletes the supply of nutrients and effectively restricts pathogen proliferation [30]. HR-mediated defence has previously been reported as the predominant defence response against *Pst* infection in resistant wheat varieties [31, 32]. As such, the observed chlorosis and regional yellowing on the leaves of the resistant Koonap cultivar could suggest an induced HR event as a defence response mechanism compared to the susceptible Gariep variety. Additionally, the possible lack of HR defence response from Gariep could further make available the necessary nutrients for the growth and proliferation of *Pst* as seen by the larger and more pronounced spores on Gariep compared to the smaller and fewer *Pst* spores from the resistant Koonap variety. Therefore, the expression of an HR in the Koonap cultivar could be a mechanism of resistance against *Pst*, and further evaluation of the biochemistry associated with the HR can lead to the discovery of markers useful in the enhancement of the defence response in *Pst*-susceptible wheat cultivars. As such, the current study utilised untargeted metabolomics analysis to evaluate the effects of pathogen infection on the plant metabolome and allowed for the comparative investigation of the time-course metabolic perturbations in a resistant vs. susceptible wheat cultivar responding to *Pst* infection.

### Differential metabolic reconfiguration of the primary metabolism

Several studies have reported on the significance of a plant’s primary metabolism in plant-microbe interactions [4, 33, 34], particularly in plant defence responses and signalling [35]. Generally, key metabolites from primary metabolism such as amino acids, organic acids, phytohormones and lipids (fatty acids) play a pivotal role in plant-pathogen interactions. For instance, high concentrations of organic acids and amino acids were characterised in bacteria (*Pseudomonas syringae*)-infected tomato plants, suggesting a significant contribution to the defence response and a cause for induced systemic resistance (ISR) [36]. Phytohormones such as auxins have been identified as key role players in pathogenesis and plant defence [37]. On the other hand, distinct amino acids and associated metabolic pathways constitute significant parts of plant immunity. For instance, the catabolism of lysine produces a non-protein, cyclic amino acid (pipecolic acid -Pip-) involved in immune signalling that amplifies plant defence responses against bacterial and fungal pathogens [38]. Additionally, aspartate-derived amino acids (lysine, methionine, threonine, and isoleucine) were found to be elevated in *P. syringae*-infected *Arabidopsis* as critical regulators of systemic acquired resistance (SAR) [39].

Tryptophan, phenylalanine and arginine were the most significantly altered amino acids identified from the study. The amino acids showed an increased accumulation in *Pst-*infected samples compared to control samples. This observation was more apparent in the *Pst*-susceptible cultivar (Gariep) compared to the *Pst*-resistant cultivar (Koonap). Arginine has been reported to play a key role in plant defence due to its functions in the glutamate-mediated central metabolism during host challenge with a pathogen [40, 41]. Arginine metabolism (oxidation) results in the formation of reactive nitrogen species (RNOs) which work in synergy with ROSs as important defence signalling molecules in the plant immune system. According to Moreau et al. [42], these molecules regulate plant defence responses ranging from HR development to hormonal and transcriptomic regulation. The treatment of post-harvest tomato fruit with L-arginine has been shown to induce resistance against *Botrytis cinerea*, which increased nitric oxide (NO), thus leading to the activation of defence enzymes such as PAL, chitinases and glucanases [43]. L-arginine is also implicit in the NO-mediated polyamine pathway in which the amino acids function as substrate molecules for the production of polyamines (putrescine, agmatine and spermine/spermidine) which play an important role in cell growth [44]. In addition, polyamine derivatives such as the cinnamamides *p*-coumaroyl agmatine, feruloyl agmatine and feruloyl putrescine (hydroxycinnamic acid amides-HCAAs) that are involved in plant defence against pathogens are further discussed later [45, 46].

Tryptophan and phenylalanine are two of the most essential compounds of the primary metabolism associated with plant defence, and these aromatic amino acids serve as the gateway to the secondary metabolism [47]. Aromatic amino acids, including tyrosine, which was detected only from Koonap, are substrates of phenolic compounds produced through the phenylpropanoid biosynthesis pathway (Figs. 4 and 5) and consequently lead to the accumulation of lignins [47–49]. The increased accumulation of these amino acids in the *Pst*-infected susceptible cultivar, particularly later in the time-course (week 3–4), could indicate the beginning stages of induced host defence. Moreover, tryptophan was flagged as a significant biomarker. In addition to gradual accumulation over time, the metabolite (trp) was detected in higher concentration in the infected susceptible plants than control uninfected (control) plants (Fig. 3B). This observation could be an indication of a delayed host response by the susceptible cultivar to pathogen infection [25].

In comparison, these amino acids showed a contrasting trend in accumulation in the *Pst-*resistant cultivar, in which the concentrations of said amino acids reduced over time. This brings forth the assumption that amino acid metabolism could be channelled to defence responses early during plant development and infection, leading to a robust and quick host defence in resistant plants compared to their susceptible counterparts. These findings correlate with previous observations as reported by Gogio et al. [50] and Mashabela et al. [25]. Additionally, the amino acid biosynthesis pathway is involved in energy metabolism. Thus, due to the already high levels of specialised phenolic compounds in the resistant plants, the aromatic amino acids could be further metabolised for energy production geared towards plant growth and development [51, 52]. Moreover, the role of tryptophan as a precursor molecule to indoles is well documented [49, 53, 54]. Although indoles and derivatives are involved in plant defence, the down-regulation of indole-3-carboxaldehyde and indole-3-acrylic acid in Gariep could further point to differences in the apparent reprogramming of primary metabolism towards the production of secondary metabolites. This observation is also in contrast to the trend seen in Koonap, where indole-3-carboxaldehyde and indole-3-acrylic acid accumulated over time as an indication of the utilisation of tryptophan to continue serving the plant’s defence metabolism.

On average, *Pst*-infected and uninfected Koonap plants showed a higher accumulation of organic acids over time compared to the susceptible Gariep plants. Organic acids, also referred to as TCA intermediates, are central to the overall metabolism of plants due to their key roles in energy and carbon metabolism [55, 56]. Organic acids are also essential for plant growth and development as mediators of the optimum photosynthetic capacity of plants and CO_2_ fixation [56]. Hence, the accumulation of organic acids in Koonap plants could point to a healthy overall cellular function of the plants in which the photosynthetic and energy metabolism machinery functions uninterrupted. In contrast, the relatively lower levels of organic acid in the susceptible Gariep cultivar could indicate pathogen proliferation which interrupts metabolite allocation. Information on the primary role of organic acids in plant defence is scarce, however, the TCA intermediates are known to facilitate biosynthesis of aromatic amino acids which can further be metabolised to produce specialised secondary defence metabolites [57–59]. Other functions include osmoregulation and the mediation of ROS and RNS generation for signal transduction. In this case, it can be speculated that the accumulation of organic acids in a plant, post-pathogen infection, helps with the defence signalling machinery for an enhanced immune response.

Furthermore, signal transduction in plants is highly regulated by lipids (fatty acids). In this regard, organic acid-mediated fatty acid biosynthesis has been reported by Zeiss et al. [60] and was speculated to reduce disease progression through lipid peroxyl production and cell membrane destruction following pathogen infection. Accumulation of fatty acids such as linolenic acid and derivatives, including dirhamnosyl linolenic acid and linolenic acid derivative isomer, were observed in both *Pst*-infected and control Koonap plants only. Precursors of linolenic acids have been reported to function as protective signalling compounds and antimicrobial metabolites against Fusarium in barley [61], and phytoalexin production in Arabidopsis [62]. Therefore, as previously mentioned, the high levels of fatty acids in Koonap could be a contributing factor to the resistance of the plant to *Pst*. Additionally, these compounds also mitigate various metabolic processes to reduce the severity of environmental stress [60, 63]. Lipids are also involved in phytohormone biosynthesis. The phytohormones JA conjugate (sulpho jasmonate) and SA were thus detected in the current study. JA and SA are pivotal compounds in plant defence and are responsible for inducing and regulating ISR and SAR. Hence, the low levels of lipids and the absence of the phytohormones from Gariep could indicate the decreased resistance to *Pst*.

### Differential metabolic reprogramming of secondary metabolism

Pathogens establish themselves within a host through the production and secretion of toxic compounds and other effector molecules which induce metabolic perturbations in the host plant. Generally, plants undergo metabolic reprogramming as a counter measure to pathogen infection and proliferation. This includes the accelerated biosynthesis of defence-related specialised secondary metabolites through several metabolic pathways [60]. As discussed above, L-arginine and proline metabolism was one of the most impacted pathways due to pathogen infection in both Koonap and Gariep plants. These metabolic pathways serve as the gateway to the production of aliphatic polyamines and phenolic acid-conjugated aryl monoamines. In this regard, arginine is decarboxylated to synthesise agmatine which is further conjugated to hydroxycinnamates or hydroxycinnamic acids (HCAs), including coumaric- and ferulic acids to produce coumaroyl agmatine and feruloyl agmatine respectively (Figs. 4 and 5). Agmatine can also undergo further hydrolysis by agmatine iminohydrolase (AIH) to produce carbamoylputrescine which is then decarboxylated to produce putrescine and lastly conjugated to form feruloyl putrescine, another member of HCAAs annotated in this study [46, 64, 65].

HCAAs are highly ubiquitous plant-specialised phenylpropanoids which are reported to play a role in plant growth and development and are generally accepted as integral components of plant tolerance to biotic and abiotic stresses [65, 66]. The accumulation of HCAAs was reported in several plant species, including *Solanum tuberosum*, *Capsicum annuum* and *Nicotiana tabacum*, as well as cereal crops such as *Zea mays*, *Avena sativa* and *T. aestivum* responding to wounding, fungal or pathogen attack [66, 67]. Recently, Knollenberg and colleagues [68] explored the performance of specialised metabolites in *Theobroma cacao* resistance against *Phytophthora* spp. The study reported a dramatic accumulation of HCAAs in the tolerant genotype (58-fold higher) compared to the susceptible counterpart. In vitro assays revealed that HCAAs inhibits the growth of the *Phytophthora* spp. pathogens, as well as the inhibition of protease and pectinase activity associated with defence in plant-pathogen interaction. The findings by Knollenberg et al. [68] correlate with those in the current study in that the HCAAs sinapoyl hydroxy agmatine, coumaroyl agmatine, feruloyl agmatine, sinapoyl putrescine and feruloyl putrescine were found in higher concentrations in the *Pst*-resistant Koonap cultivar compared to its susceptible Gariep counterpart, thus suggesting a potential functional role of these compounds in plant defence.

Figure 3 presents a striking difference in the metabolic profiles between the *Pst*-resistant Koonap and susceptible Gariep cultivars, particularly regarding the identities of the detected significant biomarkers. Several phenolic metabolites were flagged as significant biomarkers for Koonap, thereby representing metabolic reprogramming due to pathogen infection. This observation reflects the finding from our previous study Mashabela et al. [25], where Koonap cultivars were assumed to display resistant traits due to their abundant phenolic compounds. The accumulation of phenolic compounds, including flavonoid glycosides, HCAs and flavone derivatives, was observed over time from both control and *Pst*-infected samples. The accrual of these compounds points to enhanced defence capabilities of the resistant genotype. Moreover, the relative concentrations of the detected phenolics were higher in the infected plants compared to control controls. This suggests a continued production of defence metabolites in correlation to disease progression, thus maintaining plant health throughout the life cycle of the pathogen. On the other hand, a gradual decrease in several phenolic compounds was observed in control Koonap plants, pointing to the redirection of metabolism towards plant growth and development instead of the production of defence metabolites.

Phenolic compounds are of great value to the biological functions of plants, and numerous studies have concluded that the phenolic content of plants increases as a result of biotic stress such as insect feeding or pathogen infection [69]. According to Mandal et al. [70], phenolic compounds play a pivotal role in the induced plant resistant phenotype. Phenolic compounds such as flavonoids and flavone or isoflavones, including rutin, kaempferitrin, kaempferol-3-O-glucoside and schaftoside, and identified as biomarkers in this study, can act as phytoanticipins [70, 71]. The presence of phytoanticipins in plants before a challenge through pathogen infection grants the plant an enhanced defence response. When synthesised *de novo*, these compounds can further display properties of phytoalexins through extended accumulation post-pathogen infection which help inhibit the growth and proliferation of the invading pathogen.

The plant protective properties of flavonoids and derivatives were reported in sorghum (*Sorghum bicolor*) [72]. These compounds were found to be up-regulated following infection with *Burkholderia andropogonis*. The study annotated several flavones including luteolin-, naringenin- and quercetin- conjugated glycosides, as well as flavones and flavonols such as apigenin, kaempferol and quercetin glycosides which were found to exhibit antioxidant activity, inhibit fungal growth and hinder microbial invasion during the developmental stages of the plants. These metabolites have further been reported as free-radical scavengers due to their ability to function as reducing agents and metal chelating properties [73]. As such, the elevated levels of these phenolic compounds in the Koonap cultivar could provide a defensive capacity and resistance to *Pst* infection compared to the relatively low abundance from the Gariep cultivar. Nonetheless, the metabolic profiles of the Gariep cultivar showed the reconfiguration of several secondary metabolites, although inconsistent with pathogen infection or response to disease progression over time.

Overall, the results showed that the metabolic reconfigurations observed from the *Pst*-resistant and susceptible wheat cultivars were in stark contrast to each other. Due to their rich diversity in defensive specialised secondary metabolites, the resistant cultivar is capable of a more robust, faster and earlier induction of a defence response to counter the deleterious effects of pathogen infection. This allows the plant to channel resources from primary metabolism to essential functions such as energy metabolism, maintenance of homeostasis and photosynthetic machinery. On the other hand, the susceptible cultivar generally exhibited a lower abundance of phenolic compounds hence, upon pathogen infection, drastic metabolic perturbations and reconfigurations in both the primary and secondary metabolism occur which further disrupt the normal metabolic activity required for plant growth and development. This phenomenon leaves susceptible varieties vulnerable to pathogen proliferation and associated lower changes of survival.

## Conclusion

Plant-pathogen interactions can cause metabolic changes in the parties involved, and such perturbations in plants due to the elicitation of a defence response upon the perception of a pathogenic stimulus induce the activation of a cascade of defence-related gene expression as well as the production of specialised defence and defence-related metabolites. The untargeted LC-MS analysis in the current study revealed the time-dependent metabolic reprogramming in *Pst-*infected wheat cultivars. The metabolic reconfigurations spanned classes of metabolites including phenylpropanoids, flavonoids, lipids and fatty acids, amino acids, indole and derivatives, and organic acids from both primary and secondary metabolism. Amino acids such as tryptophan, tyrosine and phenylalanine were highly upregulated in the susceptible *Pst*-infected Gariep cultivar compared to the resistant Koonap counterpart. This observation was an indication of the redirection of amino acid biosynthesis towards defence metabolite biosynthesis in response to pathogen infection. In contrast, the utilisation of these amino acids was not much required for the Koonap cultivar and were thus found in lower abundance. The delayed deployment of amino acids points to the differences in the response times between the *Pst*-resistant and susceptible cultivars. The lower levels of aromatic amino acids in the resistant cultivar also suggest the continuous utilisation of these compounds to produce specialised defence metabolites. Additionally, flavonoids were the most significantly altered metabolites in the Koonap cultivar, thus presenting possible biomarkers for resistance against *Pst* infection in wheat. The identified biomarker metabolites could serve as test subjects in exogenous applications for the elicitation or enhancement of *Pst* resistance in wheat cultivars. As such, metabolic manipulation/engineering might offer an alternative to conventional plant/crop breeding strategies where, generally, only the production of specific metabolites can be modulated for plant protection against biotic stress agents.

## Experimental procedures

### Plant growth

The germination soil mixture (in 9 cm germination pots) was soaked overnight in Supafeed® 3:1:6 (46) (AECI Plant Health, Modderfontein, South Africa), a water soluble fertiliser consisting of nitrogen (N) − 155 g/kg, phosphorus (P) – 46 g/kg, potassium (K) − 267 g/kg, sulphur (S) − 4.1 g/kg, magnesium (Mg) − 3.1 g/kg, zinc (Zn) − 711 mg/kg, boron (B) − 1073 mg/kg, molybdenum (Mo) − 67 mg/kg, iron (Fe) − 765 mg/kg, manganese (Mn) − 278 mg/kg and copper (Cu) – 77 mg/kg. Wheat (*Triticum aestivum* L.) seeds (approximately 15 seeds per pot) of the *Pst*-resistant Koonap and susceptible Gariep cultivars (obtained from the Small Grain Centre, Agricultual Research Council, South Africa) were sown and germinated in the potted germination mixture (Culterra, Muldersdrift, South Africa) under controlled greenhouse conditions. The Koonap cultivar has been reported to display resistance to wheat rusts such as stem rust caused by *Puccinia graminis* f. sp. *tritici* (*Pgt*) and *Pst*, while the Gariep cultivar displays susceptibility to such wheat rusts [74, 75]. The plants were grown in temperatures ranging between 22ºC to 23ºC, watered biweekly with distilled water and a fertiliser solution made up of 5 g Supafeed^®^/L until germination and subsequent *Pst* infection as described below.

### *P. striiformis* spore multiplication, plant infection and harvesting

Dried *P. striiformis* urediniospores were obtained from the ARC-SG crop improvement division, where spore multiplication was done on *Pst*-susceptible Morocco wheat seedlings to enhance spore germination. Multiplied spores were collected from plants and prepared for inoculation. Spores were reconstituted in Soltrol-170^®^ isoparaffinic mineral oil (Chevron Phillips Chemical Company, Belgium) as a carrier at a concentration of 5 mg spores/mL (6 × 10^6^ spores/mL) before inoculation. The plant leaf blades were then pressure inoculated with the reconstituted spores, while control plants were mock-treated with only Soltrol-170^®^ mineral oil in a controlled infection using a Vacuumbrand^®^ pressure pump. The Soltrol-170 mineral oil carrier was evaporated by placing inoculated plants under direct light for 30 min. The plants were then misted with light water sprays to enhance spore germination, incubated in a dark cold room at 10–15ºC overnight, and then transferred to a glass house maintained at 15–18ºC with 12 h/12 h light/dark cycles. Infected plants were monitored for symptom development from 14 days post-inoculation (d.p.i.). Sample harvesting was done once weekly for four consecutive weeks, where three plants per sample (three biological replicates) were harvested as independent biological replicates and cryopreserved (quenched) in liquid nitrogen to prevent further metabolic and enzymatic activity. The samples were stored at -80ºC until metabolite extraction.

### Metabolite extraction and UHPLC-ESI-Q-TOF-MS data acquisition

Metabolite extraction and data acquisition were followed as described by Mashabela et al. [25]. In short, concentrated samples of treated and non-treated cultivars were reconstituted in 50% LC-grade methanol and filtered into LC-MS vials for analysis. Equal volumes of the sample aliquots were pooled together to prepare quality control (QC) samples used to assess the reliability and reproducibility of the analytical method. Sample analysis was performed in triplicate on an ultra-high-performance liquid chromatography system coupled to high-definition mass spectrometry (UHPLC-HD-MS) (Synapt G1 Q-TOF MS, Waters Corporation, Milford, MA, USA) fitted with a Waters Acquity HSS T3 C18 column (150 mm x 2.1 mm x 1.8 μm). LC separation was performed with a binary mobile phase composed of water (eluent A) and acetonitrile (eluent B) (Romil Pure Chemistry, Cambridge, UK), both with 0.1% formic acid and 2.5% isopropyl alcohol (Sigma-Aldrich, Munich, Germany), and used in gradient elution at a flow rate of 0.4 mL/min and a run time of 30 min. Eluent B ranged from 2% over the first 2.0 min, 2–90% over 2.0–25 min, 90–95% over 25–27 min, then returned from 95 to 2% over 28–30 min. Finally, the column was washed with a solution of methanol:acetonitrile isopropyl alcohol (MeOH:ACN:IPA) for regeneration. The samples were analysed from three biological replicates, and each analysed in three technical replicates such that n = 9.

MS detection was carried out in an electrospray ionisation (ESI) source in both positive and negative modes on a Waters Synapt G1 Q-TOF MS. The MS conditions were set as follows: 2.5 kV capillary voltage and 30 V sample cone voltage with 1800 V MCP detector voltage, a source temperature of 120 °C, and a 450 °C desolvation temperature. The cone gas flow was set at 50 L/h, desolvation gas flow at 550 L/h, *m/z* range of 50–1200, a 0.1 s scan time in centroid mode with interscan delay: 0.02 s, and a mass accuracy window of 0.5 Da. The MS experiment file was set up to perform both unfragmented as well as five fragmenting experiments (MS^E^) simultaneously, by increasing in-source collision energy from 3 to 50 eV to assist with subsequent structural elucidation and compound identification based on mass spectral information (MSI Level 2) [76].

### Data pre-processing and multivariate data analysis (MVDA)

Pre-processing of UHPLC-Q-TOF-MS raw data was done on MarkerLynxTM software (version 4.1, Waters Corporation, Milford, MA, USA) for both positive and negative data. MS data was processed as per the parameters outlined by Mashabela et al. [25] with minor modifications: Rt range of 0.68–26.35 min, a mass range of 100–1500 Da, a mass tolerance of 0.05 Da, and noise elimination level of 10 followed by data normalisation on MassLynx XMTM software (Waters, Manchester, UK). For multivariate data analysis (MVDA), pre-processed data matrices were exported for analysis on MetaboAnalyst 5.0 online software (www.metaboanalyst.ca) on which partial least-squares discriminant analysis (PLS-DA) and orthogonal-partial least square discriminant analysis (OPLS-DA, a supervised, binary classification method) modelling were performed. Resulting PLS-DA models reduced the dimensionality of the data to present a two-dimension graphical depiction of summarised indices from the data matrix for better visualisation and interpretation. In turn, OPLS-DA score plots were used for binary classification to reveal underlying metabolite features contributing to observed discrimination between treated and non-treated groups of data. OPLS-DA S-plots highlighted discriminant biomarkers represented by VIP scores plots. PLS-DA generally explains the difference between two or more class properties and can thus be applied to multi-group classification, while OPLS-DA is applied to expose class separations between two experimental groups.

Annotation of significant ions contributing to the variation in the metabolite profiles of the treated and non-treated cultivar varieties was performed using molecular networking tools such as MS-DIAL according to Mashabela et al. [47], and further confirmed manually using MS-based accurate mass. Level 2 MSI guidelines [76] were applied to putatively annotate metabolites based on the similarities of experimental data, including mass spectral patterns and elemental composition with reference to published literature. The *m/z* of the selected compounds were used to determine their empirical formulae and were then used to mine online databases such as the Dictionary of Natural Products (DNP), PubChem, ChemSpider to aid in compound identification. Metabolite annotation and identification was followed by biological interpretation of observed MVDA data in which annotated metabolites were used to metabolic networks from metabolic pathway analysis (MetPA) on MetaboAnalyst-powered by the Kyoto Encyclopedia of Genes and Genomes (KEGG), to visualise the impacts of metabolic reprogramming on the overall metabolism of the plants.

## Electronic supplementary material

Below is the link to the electronic supplementary material.

Supplementary Material 1

## Data Availability

The datasets used and/or analysed during the current study is available from the corresponding author on reasonable request.
