# Supplementary material for: Metabolite profiling of susceptible and resistant wheat (Triticum aestivum) cultivars responding to Puccinia striiformis f. sp. tritici infection
Source: BMC Plant Biol. 2023 Jun 1;23:293. doi: 10.1186/s12870-023-04313-9 (PMC10233866; doi:10.1186/s12870-023-04313-9)
Supplement: Supplementary file 1 — Supplementary Material 1 [file 12870_2023_4313_MOESM1_ESM.docx]

**BMC Supplementary file.**


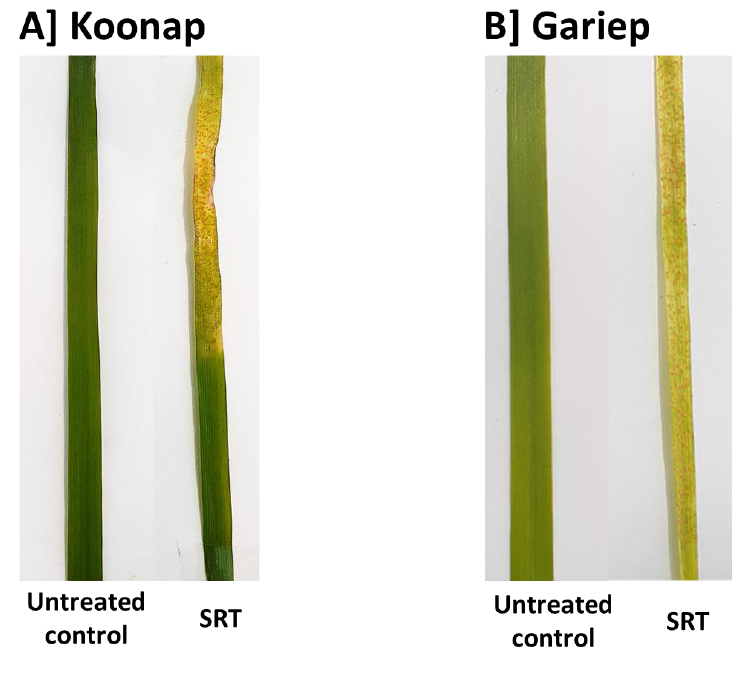


**Fig S1: Symptom development in wheat cultivars infected with *Pst***. Symptom development was monitored from 14 days post-infection. The resistant Koonap (**A**) cultivar showed considerable chlorosis and leaf yellowing at the site of infection compared to the susceptible Gariep variety (**B**). There was also a restricted development of *Pst* spores in both size and proliferation on the resistant cultivar compared to the susceptible counterpart. The observations made from the symptoms suggests the induction of an HR in the resistant cultivar as a means of defence response against pathogenic infection. SRT = *Pst*-infected.


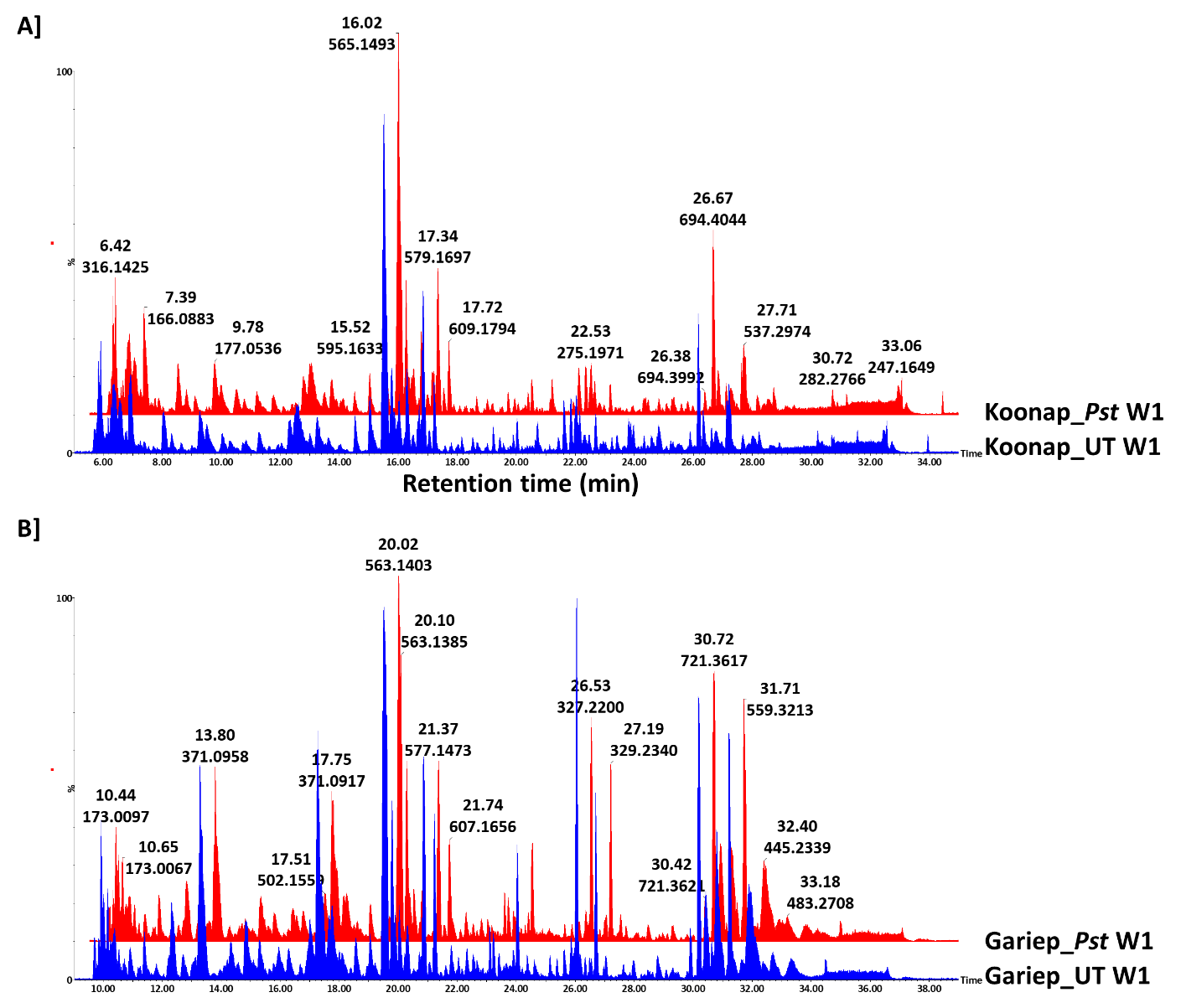


**Fig S2: ESI negative BPI MS chromatograms of *Pst*-infected and untreated (UT) wheat cultivars at week 1 post-infection**. Here, the variations in the metabolic profiles of methanol extracts from *Pst*-resistant Koonap (**A**) and susceptible Gariep (**B**) at week one post-infection are seen. The chromatograms show difference in peak populations and peak intensities. Koonap infection with *Pst* shows a relative increase in metabolites compared to the untreated samples. Surprisingly, infected Gariep samples remained relatively similar in peak population and peak intensities in comparison to the untreated controls. Moreover, *Pst*-treated Gariep showed a further decrease in some metabolites (*m*/*z* 173.0097 at Rt 10.44; 371.0958 at Rt 13.80; 577.1473 at Rt 21.37; and 327.2200 at Rt 26.53).


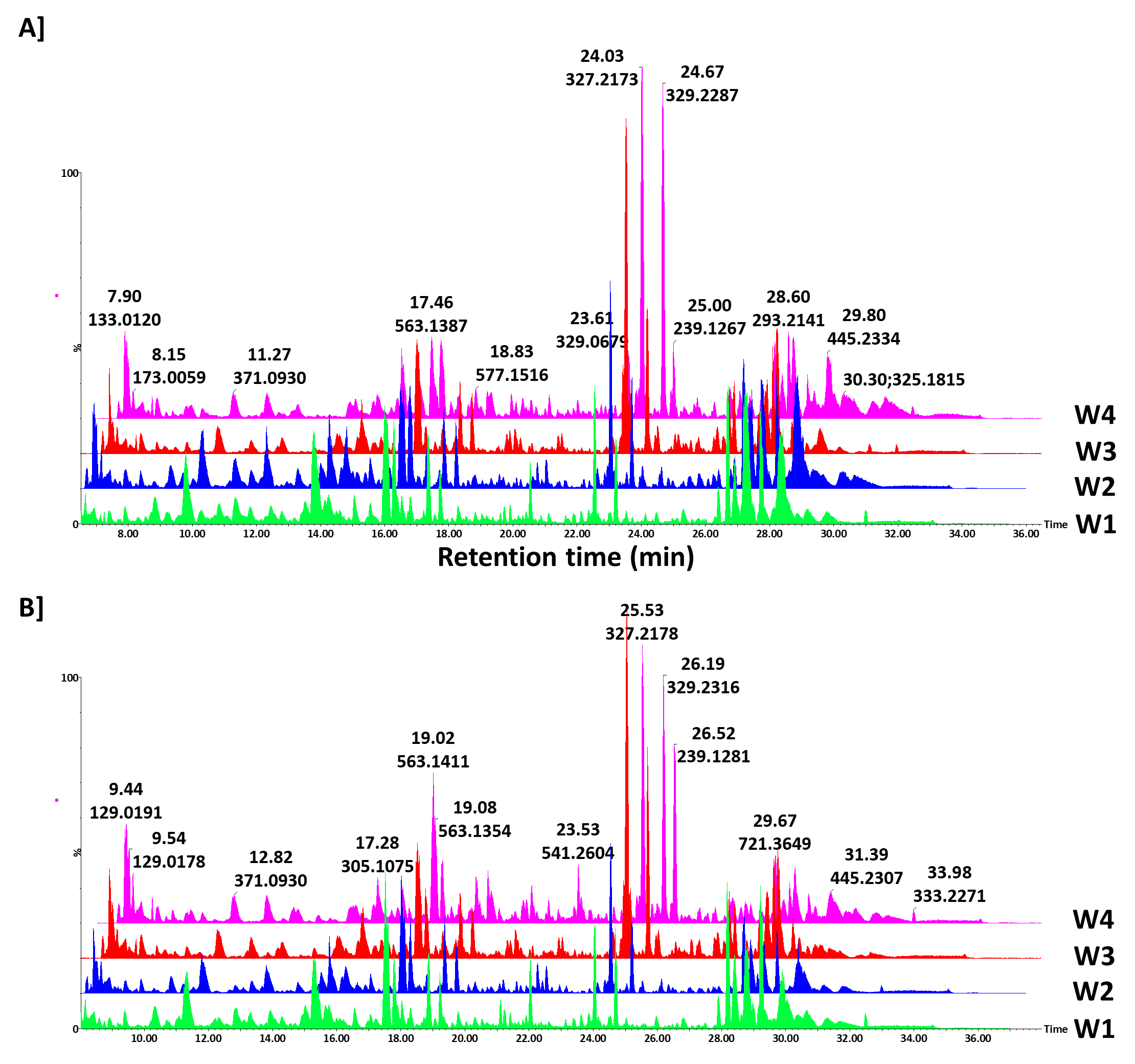

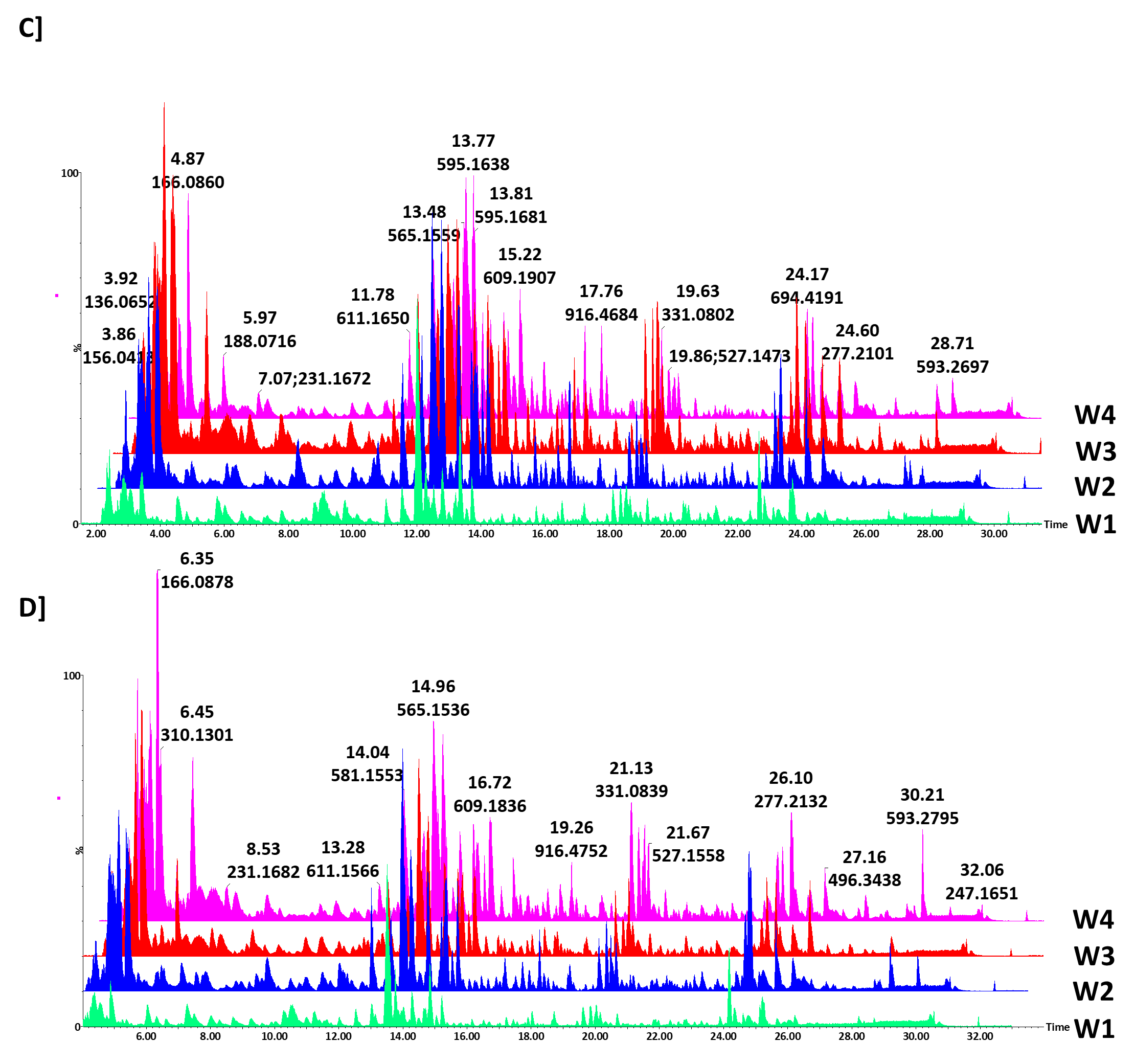


**Fig S3: ESI negative BPI MS chromatograms of *Pst*-infected (SRT) and untreated (naive) Gariep and Koonap cultivars showing metabolic changes over a four-week period**. The variations in the metabolic profiles of methanol extracts from naive (control) samples (**A**) and *Pst-*infected Gariep (**B**) as well as extracts from naive (control) samples (**C**) and *Pst-*infected Koonap (**D**) over a four-week period (W1 to W4) were observed. The BPI chromatograms show differences in peak populations and peak intensities. The majority of metabolites were shown to increase with time, while some metabolites fluctuated, thus indicating a time-dependant metabolic reprogramming. Infected samples showed a greater accumulation of metabolites from week 1 to 3 and a decrease at week 4 post-infection, while uninfected samples showed only a steady accumulation of some metabolites from week 1 to 4, with the most significant differences observed from week 3 and 4 profiles of the samples.


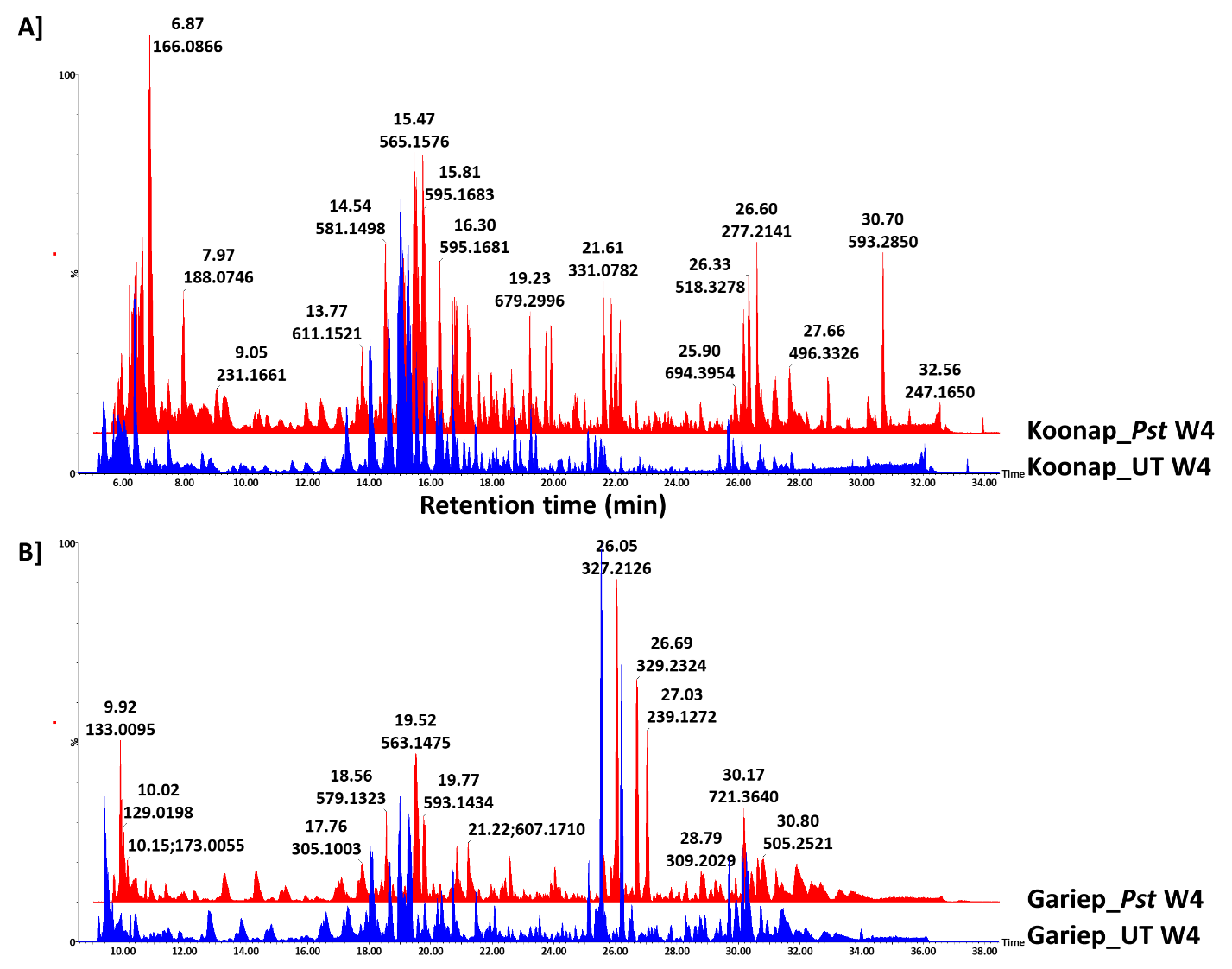


**Fig S4: ESI negative BPI MS chromatograms of *Pst*-infected and untreated (UT) wheat cultivars at week 4 post-infection**. The variations in the metabolic profiles of methanol extracts from *Pst*-resistant Koonap (**A**) and susceptible Gariep (**B**) at week four post-infection were observed. The chromatograms show difference in peak populations and peak intensities. Koonap infection with *Pst* shows a relative increase in metabolites compared to the untreated samples. Surprisingly, infected Gariep samples remained relatively similar in peak population and peak intensities in comparison to the untreated controls. Moreover, *Pst*-treated Gariep showed a further decrease in some metabolites (*m/z* 327.2126 at Rt 26.05; and 329.2324 at Rt 26.69).


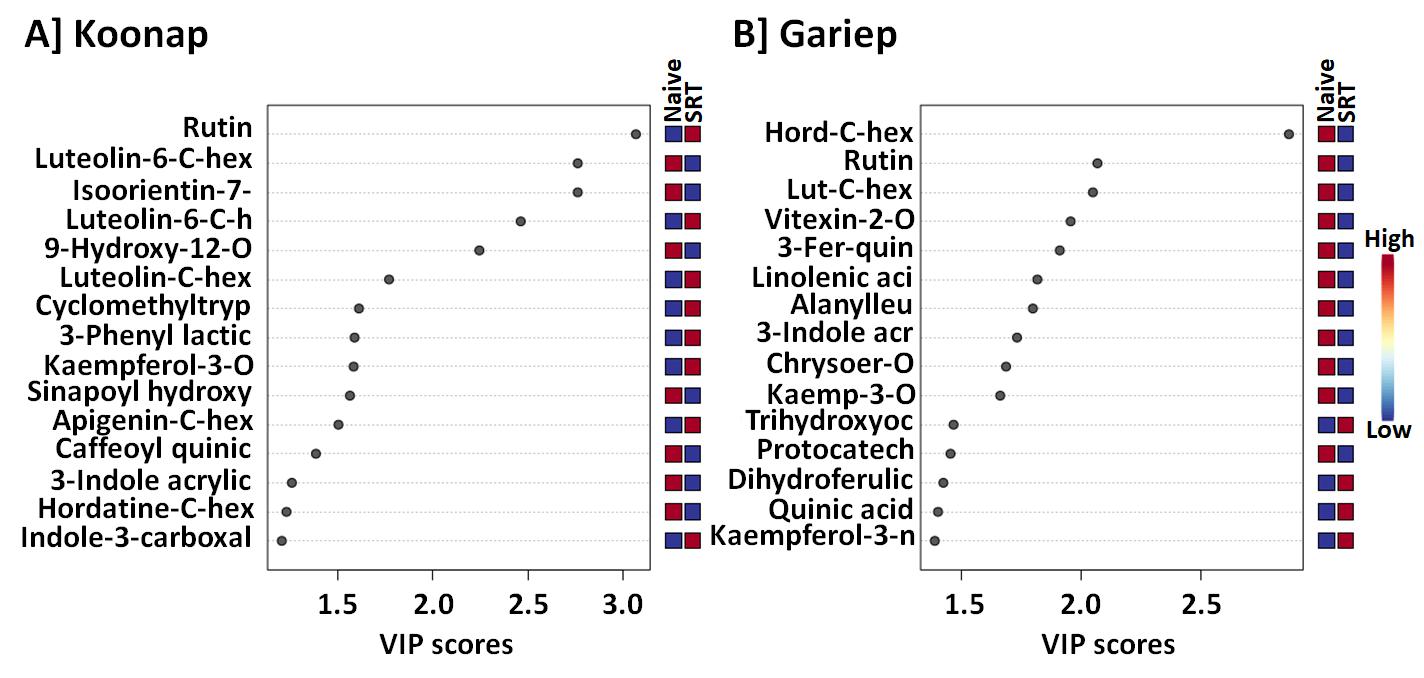


**Fig S5: VIP score-plots derived from the PLS-DA analysis**. The VIP scores display discriminant features in *Pst*-infected plant samples (SRT) compared to untreated (naive) samples for Koonap (**A**) and Gariep (**B**). Selected metabolites (VIP score ≥ 1) in infected plants were compared to those in control plants. The figures show differential up-/down-regulation of significant metabolites in infected plant compared to control (naive) plants. The data projected above were median-normalised, log transformed and *Pareto*-scaled in MetaboAnalyst.


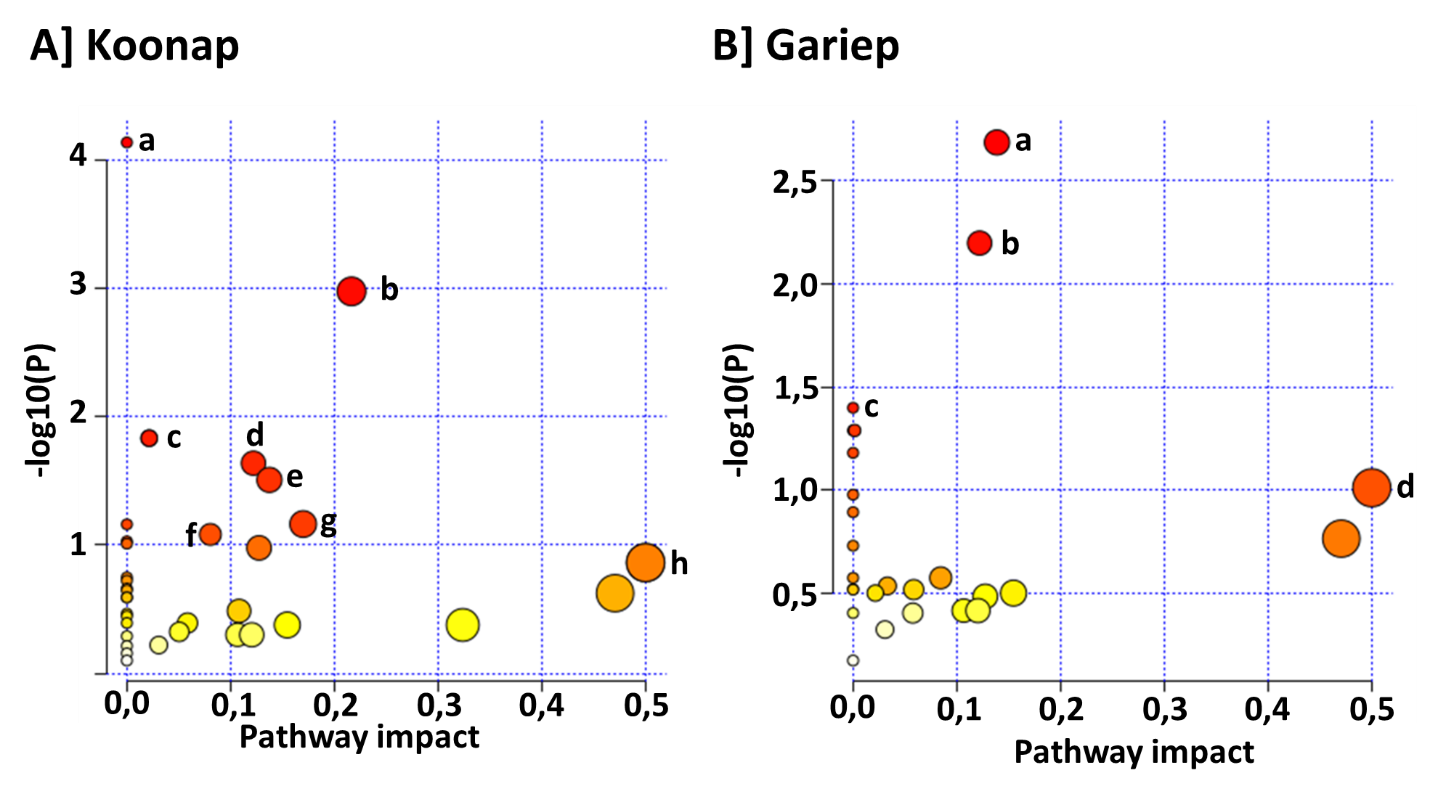


**Fig S6: MetaboAnalyst-computed pathway analysis.** Pathway view of statistically significant pathways flagged from the metabolome view based on matched metabolites. The figures show pathways matched from the annotated metabolites of Gariep and Koonap wheat cultivars. The pathways are arranged based on the *p*-value (y-axis), which indicates the pathway enrichment analysis, and pathway impact values (x-axis) representing pathway topology analysis. The node colour of each pathway is determined by the *p*-value (red = lowest *p*-value and highest statistical significance), and the node radius (size) is based on the pathway impact factor, with the biggest indicating the highest impact. The impacted pathways in Koonap **(A)** included **a)** aminoacyl tRNA biosynthesis, **b)** arginine and proline metabolism, **c)** phenylalanine, tyrosine and tryptophan biosynthesis, **d)** phenylpropanoid biosynthesis, **e)** glyoxylate and dicarboxylate biosynthesis, **f)** TCA cycle, **g)** arginine biosynthesis, and **h)** phenylalanine biosynthesis. The most significantly impacted pathways in Gariep **(B)** are **a)** arginine and proline metabolism, **b)** phenylpropanoid biosynthesis, **c)** phenylalanine, tyrosine and tryptophan biosynthesis, and **d)** phenylalanine metabolism.

**Table S1: Summary of the annotated, putatively identified metabolites (MSI-L2)**.

| **No.** | **Compound** | **Chemical Formula** | **Rt (min)** | ***m/z*** | **Adduct** | **Fragments (*m/z*)** |
| --- | --- | --- | --- | --- | --- | --- |
| **1** | L-Arginine | C6H15N4O2 | 0.75 | 175.1025 | [M+H]+ | 158, 130, 118, 116 |
| **2** | Choline | C5H14NO | 0.86 | 104.1062 | [M+H]+ | 60, |
| **3** | Adenine | C5H5N5 | 0.92 | 136.0658 | [M+H]+ | 118, |
| **4** | Citraconic acid | C5H6O4 | 0.98 | 129.0194 | [M-H]- | 85 |
| **5** | Malic acid | C4H6O5 | 1.04 | 133.0127 | [M-H]- | 191, 115 |
| **6** | Quinic acid | C7H12O6 | 1.24 | 191.0182 | [M-H]- | 173, 111 |
| **7** | Aconitic acid | C6H6O6 | 1.28 | 173.0889 | [M-H]- | 129, 117, 85 |
| **8** | Indoline | C8H9N | 1.85 | 120.0814 | [M+H]+ | 103, |
| **9** | L-Phenylalanine | C9H11NO2 | 2.03 | 164.0706 | [M-H]- | 147, 129, 103 |
| **10** | Protocatechuic acid-4-glucoside | C13H16O9 | 2.18 | 315.0755 | [M-H]- | 153, 108 |
| **11** | Pantothenic acid | C9H17NO5 | 2.23 | 220.1169 | [M+H]+ | 185, 116, 90 |
| **12** | Alanylleucine | C9H18N2O3 | 2.54 | 203.1404 | [M+H]+ | 157, 132, 86 |
| **13** | Indole-3-carboxaldehyde | C9H7NO | 2.98 | 146.0616 | [M+H]+ | 118, 91 |
| **14** | 3-(3,4,5-Trihydroxyphenyl) propanoic acid | C9H10O5 | 3.2 | 197.0412 | [M-H]- | 153, 151, 138, 123, 109 |
| **15** | L-Valine | C5H11NO2 | 3.35 | 118.0868 | [M+H]+ | 70, 55 |
| **16** | L-Tryptophan | C11H12N2O2 | 3.37 | 205.0968 | [M+H]+ | 188, 146, 118 |
| **17** | L-Leucine | C6H13NO2 | 3.87 | 132.0865 | [M+H]+ | 86, 69 |
| **18** | L-Glutamic acid | C5H9NO4 | 4.19 | 148.0580 | [M+H]+ | 130, 84, 72 |
| **19** | Coumarin | C9H6O2 | 4.19 | 147.0470 | [M+H]+ | 103, 91 |
| **20** | L-Tyrosine | C9H11NO3 | 4.24 | 182.0807 | [M+H]+ | 165, 136, 147, 123, 119, 91 |
| **21** | Feruloyl agmatine | C15H22N4O3 | 4.71 | 307.1698 | [M+H]+ | 307, 273, 177, 145 |
| **22** | Ferulic acid | C10H9O3 | 4.82 | 177.0552 | [M+H]+ | 145, 117, 89 |
| **23** | Cyclomethyltryptophan | C12H12N2O2 | 5.08 | 217.0977 | [M+H]+ | 144, |
| **24** | Feruloyl putrescine | C14H20N2O3 | 5.19 | 265.1552 | [M+H]+ | 248, 177, 145 |
| **25** | Tri (ethyl carbonate) | C16H18O11 | 5.37 | 385.0782 | [M-H]- | 297, 89 |
| **26** | *N-*Feruloyl spermaidine | C17H28N3O3 | 5.77 | 322.1654 | [M+H]+ | 321, 177, 163, 146, 117, 89 |
| **27** | 3-Indole acrylic acid | C11H9NO2 | 5.8 | 188.0712 | [M+H]+ | 146, 144, 118, 102, 72 |
| **28** | 3-Feruloyl quinic acid | C17H20O9 | 5.91 | 367.0970 | [M-H]- | 351, 219, 193, 178, 134, 102 |
| **29** | 3-Feruloyl quinic acid isomer | C17H20O9 | 5.92 | 367.1045 | [M-H]- | 193, 134 |
| **30** | Coumaroyl agmatine | C14H20N4O2 | 6.24 | 277.1623 | [M+H]+ | 260, 218, 147, 145, 131, 114 |
| **31** | 2-O-Glucosyl-7-methoxy-1,4(2H)-benzoxazin-3-one (HMBOA + O-Hex) | C15H19NO9 | 7.17 | 356.0978 | [M-H]- | 300, 194, 166, 138 |
| **32** | Saccharide compound | C16H20O10 | 7.29 | 371.0976 | [M-H]- | 249, 231, 121, 113 |
| **33** | Nicoblumin | C25H42O13 | 7.31 | 549.2547 | [M-H]- | 387, 227 |
| **34** | *N-*Feruloyl agmatine | C15H22N4O3 | 8.05 | 307.1725 | [M+H]+ | 290, 248, 247, 178, 177, 145, 117, 114, 95 |
| **35** | *N*-Acetyl-aspartyl glutamic acid | C11H16N2O8 | 8.06 | 303.0820 | [M-H]- | 303, 96 |
| **36** | 4-acetyl-2(3H)-Benzoxazolone (ABOA) | C9H7NO3 | 8.11 | 178.0497 | [M+H]+ | 150, 122, 95, 86 |
| **37** | Sinapoyl hydroxyagmatine | C16H24N4O5 | 8.59 | 351.1268 | [M-H]- | 249, 101 |
| **38** | Luteolin-6-C-hexoside-O-hexoside | C27H30O16 | 8.88 | 611.1612 | [M+H]+ | 449, 451, 413, 329 |
| **39** | Dihydroferulic acid 4-O-glucuronide | C16H20O10 | 9.25 | 371.0978 | [M-H]- | 195, 175 |
| **40** | Luteolin-C-hexoside-C-pentoside Isomer | C26H28O15 | 9.61 | 579.1350 | [M-H]- | 489, 459, 399, 369, 339 |
| **41** | 1-O-Sinapoyl-β-D-glucose | C17H22O10 | 9.81 | 385.1135 | [M-H]- | 223, 164 |
| **42** | 8-Arabinosyl-6-glucosylluteolin | C26H28O15 | 9.96 | 579.1332 | [M-H]- | 561, 489, 459, 399 |
| **43** | Kaempferol-3-O-galactoside-7-O-rhamnoside | C27H30O15 | 10.11 | 593.1525 | [M-H]- | 447, 283 |
| **44** | Luteolin-6-C-hexosyl-O-hexoside | C27H30O16 | 10.15 | 611.1700 | [M+H]+ | 593, 575, 545, 461, 431, 413, 395, 383, 353, 329, 299 |
| **45** | Kaempferol-3-O-rutinoside | C27H30O15 | 10.45 | 593.1498 | [M-H]- | 447, 300, 285, 284 |
| **46** | Isovitexin-7-O-glucoside | C27H30O15 | 10.45 | 593.1506 | [M-H]- | 473, 431, 341, 311, |
| **47** | Quercetin-3-O-pentosyl-pentoside | C25H26O15 | 10.46 | 565.1477 | [M-H]- | 447, 309, 285 |
| **48** | Hordatine-C-hexose isomer I | C44H33O16 | 10.52 | 771.2019 | [M-H]- | 771, 609, 593, 503, 473 |
| **49** | Rutin | C27H30O16 | 10.54 | 609.1469 | [M-H]- | 593, 447, 309, 285 |
| **50** | Apigenin C-hexoside-C-pentoside | C26H28014 | 10.6 | 565.1557 | [M+H]+ | 547, 529, 511 |
| **51** | Schaftoside | C26H27014 | 10.61 | 565.1670 | [M+H]+ | 427, 409,379, 337, 325 |
| **52** | Sinapoyl aldehyde | C11H12O4 | 10.84 | 209.0797 | [M+H]+ | 181, 177, 121 |
| **53** | Isoschaftoside | C26H28O14 | 10.86 | 563.1401 | [M-H]- | 473; 353; 325 |
| **54** | Luteolin-C-hexoside-O-deoxyhexoside | C27H30O15 | 10.86 | 595.1663 | [M+H]+ | 449, 431, 383, 353, 329, 299 |
| **55** | Luteolin-6-C-glucoside | C21H20O11 | 10.86 | 447.0917 | [M+H]+ | 431, 413, 353, 329, 299 |
| **56** | Kaempferol-3-neohesperidoside | C27H30O15 | 10.87 | 595.1775 | [M+H]+ | 449, 299, 229, |
| **57** | Iso-orientin | C21H20O11 | 11.16 | 447.0927 | [M-H]- | 429, 357, 327, 285 |
| **58** | Apigenin-6-C-glucosyl-8-C-(2''-O-dihydroferuloyl)-glucoside | C34H28O21 | 11.37 | 771.2049 | [M-H]- | 593, 503, 473, 383 |
| **59** | Kaempferol-3-O-glucoside | C21H20O11 | 11.4 | 447.0907 | [M-H]- | 285, 284, 255, 227 |
| **60** | 4-Coumaric acid | C9H8O3 | 11.44 | 165.0531 | [M+H]+ | 145, 123, 119, 103, 89, 69 |
| **61** | Loliolide | C11H16O3 | 11.58 | 197.1178 | [M+H]+ | 179, 161, 133, 107 |
| **62** | 6,8-di-C-glucosyl apigenin | C27H30O15 | 11.62 | 593.1506 | [M-H]- | 575, 473, 372 |
| **63** | Chrysoeriol-O-hexoside-C-hexoside | C28H32016 | 11.78 | 625.1769 | [M+H]+ | 463, 445, 427, 409, 397, 367, 343, 313 |
| **64** | Apigenin-6-C-glucoside | C21H20O10 | 11.84 | 433.1135 | [M+H]+ | 415, 397, 379, 349, 337, 313, 283 |
| **65** | Kaempferol-3-O-rhamnoside-7-O-rhamnoside | C27H30O14 | 11.91 | 577.1519 | [M-H]- | 431 |
| **66** | Vitexin-2''-O-rhamnoside | C27H30O14 | 11.93 | 579.1659 | [M+H]+ | 433, 415, 397, 367, 313, 204 |
| **67** | Isovitexin | C21H20O10 | 11.93 | 433.1105 | [M+H]+ | 415, 397, 367, 313, 204 |
| **68** | Kaempferitrin | C27H30O14 | 12.13 | 577.1557 | [M-H]- | 563, 453, 431, 413, 355, 341, 293, 283 |
| **69** | Chrysoeriol-O-deoxyhexoside-C-hexoside | C28H32O15 | 12.28 | 609.1819 | [M+H]+ | 463, 445, 427, 409, 397, 367, 343, 313 |
| **70** | Chrysoeriol-6-C-glucoside | C22H22O11 | 12.28 | 463.1240 | [M+H]+ | 445, 427, 409, 397, 391, 379, 367, 343, 313 |
| **71** | Chrysoeriol-O-hexoside | C22H22O11 | 12.29 | 463.1240 | [M+H]+ | 301 |
| **72** | Diosmetin-7-rutinoside | C28H32O15 | 12.31 | 609.1820 | [M+H]+ | 463, |
| **73** | 3-Phenyl lactic acid | C19H10O3 | 12.37 | 165.0552 | [M-H]- | 147, 119, 103, 73, 59 |
| **74** | *p*-Coumaraldehyde | C9H8O2 | 12.42 | 147.0430 | [M-H]- | 119,103, 59 |
| **75** | Isoorientin-7-O-glucoside | C27H30O16 | 13.01 | 611.1570 | [M+H]+ | 449, 431, 383, 353, 329, 299 |
| **76** | Luteolin-C-[pentosyl-O-(feruoyl-O-hexoside)] | C36H36O18 | 13.02 | 757.2074 | [M+H]+ | 449, 431, 413, 309, 177 |
| **77** | Chrysoeriol-O-hexoside C-(O-feruoyl-hexoside) | C38H40O19 | 13.02 | 801.230 | [M+H]+ | 463, 445, 117 |
| **78** | Tricin-7-O-deoxyhexosyl-O-hexoside | C29H34O16 | 13.22 | 639.1925 | [M+H]+ | 493, 331 |
| **79** | Tricin-7-O-hexoside | C23H24O12 | 13.57 | 493.1330 | [M+H]+ | 331 |
| **80** | Gallic acid monohydrate | C9H16O4 | 13.71 | 187.0951 | [M-H]- | 169, 125 |
| **81** | Caffeoyl | C9H7O3 | 13.85 | 163.1123 | [M+H]+ | 145, 135, 117, 89 |
| **82** | Isovitexin-6''-O-glucoside | C27H30O15 | 14.11 | 595.1630 | [M+H]+ | 595, 433, 415, 367, 337, 313, 283 |
| **83** | Tricin-7-O-hexoside malonylated | C26H26O15 | 14.55 | 577.130 | [M+H]+ | 493, 331 |
| **84** | Luteolin-O-(O-caffeoyl-hexoside) C-hexoside | C36H36O19 | 15.58 | 773.1929 | [M+H]+ | 449, 431, 329 |
| **85** | (10E,15Z) 9,12,13-trihydroxyoctadeca-10,15-dienoic isomer I | C18H32O5 | 17.27 | 327.2171 | [M-H]- | 229, 211, 183, 171, 113 |
| **86** | Trihydroxyoctadecenoic acid | C18H34O5 | 17.93 | 329.2296 | [M-H]- | 229, 211 |
| **87** | 9-Hydroxy-12-oxo-10(E),15(Z)-octadecadienoic acid isomer I | C18H30O4 | 18.07 | 309.2078 | [M-H]- | 291, 197 |
| **88** | (10E,15Z)-9,12,13-Trihydroxy-10,15-octadecadienoic acid | C18H32O5 | 19 | 327.2015 | [M-H]- | 307, 291, 227, 213, 209, 185, 155 |
| **89** | Linolenic acid derivative isomer III | C30H38O3 | 20.05 | 445.2310 | [M-H]- | 311, 293, 277 |
| **90** | 12-Oxo-phytodienoic acid (12-OPDA) | C18H28O3 | 20.12 | 291.1910 | [M-H]- | 273, 247, 209, 165 |
| **91** | OPDA conjugate isomer II | C18H30O4 | 20.14 | 309.1913 | [M-H]- | 291, 273, 247, 209, 165 |
| **92** | *N*-Sinapoyl putrescine | C15H22N2O4 | 20.76 | 295.2245 | [M+H]+ | 207, 175, 147, 119 |
| **93** | 9-Hydroxy-12-oxo-10(E),15(Z)-octadecadienoic acid isomer II | C18H32O3 | 20.96 | 295.2256 | [M-H]- | 291, 247, 165 |
| **94** | Linolenic acid | C18H30O2 | 21.12 | 277.2149 | [M-H]- | 253, 235, 221, 197, 183, 179, 161, 113, 89 |
| **95** | Linolenic acid derivative isomer I | C33H56O14 | 21.13 | 675.3592 | [M-H]- | 415, 397, 277, 235, 89 |
| **96** | Linolenic acid derivative isomer II | C33H56O14 | 21.42 | 675.3744 | [M-H]- | 415, 397, 277, 235, 89 |
| **97** | Arachidonic acid | C20H32O2 | 21.66 | 305.2474 | [M+H]+ | 121 |
| **98** | Monogalactosylmonoacylglycerol (MGMG 18:3) | C27H46O9 | 22.28 | 559.3075 | [M-H]- | 513, 277, 253, 235 |
| **99** | Dirhamnosyl linolenic acid | C28H48O11 | 22.48 | 559.3118 | [M-H]- | 277 |
| **100** | Hydroxy octadecadienoic acid | C18H32O3 | 22.69 | 295.2273 | [M-H]- | 277, 233, 195 |
